# Enigmatic intractable Epilepsy patients have antibodies that bind glutamate receptor peptides, kill neurons, damage the brain, and cause Generalized Tonic Clonic Seizures

**DOI:** 10.1007/s00702-024-02855-2

**Published:** 2025-02-11

**Authors:** Rhoda Olowe Taiwo, Hadassa Sterm Goldberg, Nili Ilouz, Prince Kumar Singh, Tawfeeq Shekh-Ahmad, Mia Levite

**Affiliations:** 1 Department of Pharmaceutics, Faculty of Medicine, The Institute for Drug Research, School of Pharmacy, The Hebrew University, Ein Karem, 91120 Jerusalem, Israel; 2 Sackler Faculty of Medicine, Tel Aviv University, Tel Aviv, Israel; 3 Institute of Pediatric Neurology, Epilepsy Center, Schneider Children’s Medical Center, Petah Tiqva, Israel; 4 Faculty of Medicine, The Hebrew University, Ein Karem, 9112102 Jerusalem, Israel; 5 Goldyne Savad Institute of Gene Therapy, Hadassah Hebrew University Hospital, 9112001 Jerusalem, Israel

**Keywords:** Epilepsy, Autoimmune Epilepsy, General tonic clonic seizures, Glutamate receptor antibodies, Glutamate receptors, GluR3B antibodies, AMPA-R antibodies, NMDA-R antibodies

## Abstract

Epilepsy affects 1–2% of the world population, is enigmatic in 30% of cases, and is often intractable, unresponsive to antiepileptic drugs, and accompanied by cognitive, psychiatric and behavioral problems. Tests for Autoimmune Epilepsy are *not* performed routinely, and limited to passive diagnosis of known autoimmune antibodies, without essential functional tests to reveal active pathogenic antibodies. We investigated two young Epilepsy patients with different Epilepsy characteristics, repeated intractable seizures, and enigmatic etiology. We suspected Autoimmune Epilepsy. We found that both patients have elevated IgG antibodies, and three types of glutamate receptor antibodies, to: AMPA-GluR3**B**, NMDA-NR1 and NMDA-NR2 peptides. In contrast, they lack autoantibodies to: LGI1, CASPR2, GABA-RB1, Amphiphysin, CV2, PNMA1, Ri, Yo, Hu, Recoverin, Soxi and Titin. IgG antibodies of both patients bound and killed human neural cells In vitro. Moreover, In vivo video EEG studies in naive rats revealed that patient’s IgG antibodies, infused continually into rat brain, bound neural cells in the hippocampus and cortex, caused neural loss in these brain regions, and induced recurrent Generalized Tonic Clonic Seizures. We assume they can do so also in the patient’s brain. This is the first model of human Autoimmune Epilepsy in rats. It can serve for discovery of patient’s pathogenic antibodies, and drug development. Tests for autoimmune antibodies that bind glutamate receptor peptides, and functional diagnostic tests, are obligatory in all enigmatic intractable Epilepsy patients. Current diagnosis of Autoimmune Epilepsy is insufficient! If pathogenic antibodies are found, intractable patients must receive available, suitable and potentially life-changing immunotherapies for Autoimmune Epilepsy.

**Supplementary Information:**

The online version contains supplementary material available at 10.1007/s00702-024-02855-2.

## Introduction

### Epilepsy

There are dozens of types of Epilepsy, affecting approximately 1–2% of the world population. Beyond its high prevalence, Epilepsy is a very difficult disease for some patients for the following reasons. First, in approximately 30% of cases, the etiology of the disease is unknown, after ruling out genetic mutations, severe injury, and several other events or factors that can cause seizures. Without knowing the etiology, tailoring an adequate personal treatment is of course very problematic, empiric, and even impossible. Second, and in line with the unknown etiology, approximately 30% of patients are unresponsive to anti-epileptic drugs (AEDs), leading to uncontrolled recurrent seizures. Intractable Epilepsy often leads to multiple seizures daily or weekly, lasting for many years. In addition to the seizures, many intractable patients suffer from other health problems: cognitive, behavioral, and psychiatric problems, as well as from other physical and social problems, which altogether make their lives miserable.

This situation makes it very difficult for learning, education, specialization, maintaining social and professional relationships, and for finding appropriate employment for patients with uncontrolled Epilepsy. In addition, families of such Epilepsy patients have very difficult lives too.

### Autoimmune Epilepsy

The term and disease ‘Autoimmune Epilepsy’ was first introduced in 2002 in a Nature Immunology paper [(Levite 2002) by ML, last author in the present study] based on a small number of pioneering In vitro and In vivo studies (Rogers et al. 1994; Twyman et al. 1995; Andrews and McNamara 1996; He et al. 1998; Levite et al. 1999; Levite and Hermelin 1999), that altogether showed that some Epilepsy patients have autoimmune antibodies, mainly Glutamate receptor (GluR) antibodies, that bind and activate by themselves Glutamate receptors (thus acting as Glutamate receptor agonists), cause excitotoxicity and neuropathology, and may induce or facilitate seizures.

Since then, Glutamate receptor antibodies and various other types of autoimmune antibodies have been found in the serum and/or cerebrospinal fluid (CSF) of subpopulations of Epilepsy patients suffering from seizures and/or encephalitis (Levite and Goldberg 2021).

These include autoimmune antibodies to the following self-antigens: Glutamate/AMPA-GluR3, Glutamate/NMDA-NR1, Glutamate/NMDA-NR2, GAD-65, GABA-R, GLY-R, VGKC, LGI1, CASPR2, β2 GP1 and few others. Some of these autoimmune antibodies were shown already to be undoubtedly pathogenic and induce on their own profound neural damage in experimental systems In vitro and In vivo (summarized in Levite and Goldberg (2021)).

For a summary, review, analysis, discussion, and new insights on ‘Autoimmune Epilepsy’, the readers are referred to Levite and Goldberg (2021). In addition, for the individual papers on the presence of GluR antibodies in Epilepsy patients, and their pathological effects In vitro and in animal models In vivo, readers are referred to the following papers (Levite 2002, 2014; Andrews and McNamara 1996; He et al. 1998; Levite et al. 1999, 2020; Levite and Hermelin 1999; Ganor et al. 2004, 2005a, b, 2014; Whitney and McNamara 2000; Wiendl et al. 2001; Wiendl et al. 2001; Mantegazza et al. 2002; Cohen-Kashi Malina et al. 2006; Levite and Ganor 2008; Solaro et al. 2006; Goldberg-Stern et al. 2014).

Despite increased awareness of ‘Autoimmune Epilepsy’, and in specific of the possibility that autoimmune antibodies can by themselves induce and/or promote epileptic seizures and additional neuropathological effects, in most countries and hospitals, patients with intractable and enigmatic Epilepsy are still lacking routinely comprehensive and suitable active diagnostic tests for ‘Autoimmune Epilepsy’. This situation prevents appropriate diagnosis of ‘Autoimmune Epilepsy’, and appropriate immunotherapy for patients that suffer from this disease. Moreover, even when clinicians suspect ‘Autoimmune Epilepsy’ and send the patient’s serum and/or CSF for diagnosis, the common diagnosis of ‘Autoimmune Epilepsy’ is limited to a number of passive In vitro diagnostic tests, mostly by Enzyme-linked immunosorbent assays (ELISA), which have two major drawbacks. First, they can only detect the presence of some already known and suspected autoimmune antibodies (those found so far in some other autoimmune neurological diseases). Second, they only measure the extent of passive binding of the patient’s serum antibodies to the respective already known autoantigenic proteins, or to cells that express them.

Thus, in the present situation, the most important *active functional tests* are missing. These needed tests are those that can reveal if the patient has active pathogenic autoimmune antibodies (regardless of their specificity, and even without known their antigenic target) that harm living neural cells/tissue In vitro and/or In vivo, induce seizures, and impair normal neurological functions. We argue that such functional tests to reveal active functional pathogenic antibodies should be performed routinely in any patient that experience recurrent seizures, especially in cases when the patient suffers also from cognitive, psychiatric and behavioral problems.

We further argue that such tests must be performed even when the passive diagnosis does not reveal the presence of well-known anti-neural autoimmune antibodies in patient’s serum and CSF. Finally, we argue that such functional tests should be preferably performed using a sample of the patient’s purified IgG’s, not the patient’s whole serum.

### Glutamate and glutamate receptor antibodies

Glutamate is the major excitatory neurotransmitter in the nervous system. Excess glutamate, and/or abnormally high neural excitation and/or over-activation of Glutamate receptors cause excitotoxicity and lead to outbursts of seizures and multiple other pathologies.

Several types of Glutamate receptor antibodies have been found so far in patients with several neurological diseases, and most of them can contribute directly or indirectly to various neuropathologies, including Epilepsy (for reviews on Glutamate receptor antibodies, readers are referred to Levite 2014; Levite and Goldberg 2021), and to various types of behavior abnormalities (Goldberg-Stern et al. 2014; Ganor et al. 2014; Scheggia et al. 2021).

Interestingly, the GluR3**B** type of Glutamate receptor antibodies has unique ability to activate Glutamate receptor of the AMPA GluR3 type (Twyman et al. 1995; Levite et al. 1999; Cohen-Kashi Malina et al. 2006; Carlson et al. 1997). The GluR3**B** antibodies can on their own activate both homomeric and heteromeric GluR3-receptor ion channels without the requirement of neuronal, glial, or blood ancillary molecules (Cohen-Kashi Malina et al. 2006), and cause excitotoxicity (Levite et al. 1999). By doing so these antibodies mimic excess Glutamate and can contribute to an outburst of seizures. (Levite et al. 1999). The NMDA receptor antibodies are also pathological and can contribute directly or indirectly to multiple neuropathologies, primarily to NMDA encephalitis (Dalmau et al. 2019), but also to Epilepsy (Levite 2014).

Recently, GluR3**B** antibodies were found in 70 out of 193 (36.3%) patients with Epilepsy and in higher levels in drug-resistant seizures (Lai et al. 2022). The GluR3**B** antibodies were found in the patient's serum and CSF. All the findings of this study showed that GluR3**B** antibodies are a biomarker for drug-resistant Epilepsy in patients with focal to bilateral tonic clonic seizures (Lai et al. 2022). In past years, we have been studying ‘Autoimmune Epilepsy’ in young patients with intractable Epilepsy of different origins and types, and revealed a handful of evidence for their presence and pathological effects (Levite et al. 1999, 2020; Levite and Hermelin 1999; Levite and Goldberg 2021; Cohen-Kashi Malina et al. 2006; Goldberg-Stern et al. 2014; Levite 2014; Ganor et al. 2014, 2005c). But we, and to the best of our knowledge no one else, found any unequivocal solid and direct evidence that Epilepsy patients’ purified antibodies can on their own induce repeated seizures, especially Generalized tonic clonic seizures (GTCS) in naïve animals. For reminder, GCTS, formerly known as grand mal seizure, are defined as seizure that have a tonic phase followed by clonic muscle contractions.

In this present paper on ‘Autoimmune Epilepsy’, we performed multilevel In vitro and In vivo investigations on two young patients with severe, uncontrolled, and enigmatic Epilepsy.

Our research revealed multiple novel findings, illustrated schematically in the Graphical Abstract (Fig. 13). Altogether our findings support our working hypothesis and show that the two Epilepsy patients we studied suffer from ‘Autoimmune Epilepsy’.

## Results

### The two intractable Epilepsy patients whose antibodies we investigated in this study

This study focuses on two young intractable Epilepsy patients called IE-3 and IE-15 throughout the article. These patients are described above in the ‘Materials’, and additional clinical information related to them is summarized in Tables 1 and 2.

**Table 1:** Basic information about the Epilepsy patients studied in the current ‘Autoimmune Epilepsy’ research

Epilepsy patient coded name and no. in our records	Date of birth	Epilepsy type	Etiology	Special education	Mental retardation	Anti epileptic drugs (AED’s)				
						AED 1	AED 2	AED 3	AED 4	AED 5
IE-3	25/7/1998	Myoclonic	Unknown	Yes	Yes	ZARONTIN	KD	DEPALEPT	KEPRA	VNS
IE-15	2/1/2003	Focal	Encephalitis	No	No		CLONEX	LACOSAMIDE	TEGRETOL	KEPRA

*VNS* Vagal Nerve Stimulation

**Table 2:** Diagnostic tests to determine the etiology of Epilepsy, performed on the two patients IE-3 and IE-15 studied in the current research

Diagnostic test	Epilepsy patient IE-3	Epilepsy patient IE-15
BLOOD		
Complete blood count	Normal	Normal
Full chemistry	Normal	Normal
Thyroid function	Normal	Normal
B12	Normal	Normal
Folic acid	Normal	Low
Lactate	Normal	Normal
Pyruvate	Normal	Normal
Amino acid	Normal	Normal
Very long chain fatty acids	Normal	Normal
Celiac screen	Normal	Normal
Porphyria screen	Normal	Normal
Rheumatic panel	Not done	Normal
Ceruloplasmin	Not done	Normal
URINE		
Organic acid	Normal	Normal
24-h copper	Not done	Normal
CSF	Normal	Normal
Cells, protein, glucose, infectious organisms	Normal	Normal
FUNDOSCOPY	Normal	Normal
IMAGING		
Brain MRI	Moderate atrophy	Mild atrophy
GENETICS		
Epilepsy panel	Negative	Negative
Exome	Negative	Negative
Specific genes for PME	Negative	Not done
BIOPSY		
Muscle	Normal	Not done
Conjunctiva	Normal	Not done

*CSF* cerebrospinal fluid, *PME* progressive myoclonic epilepsies

**Fig. 1:**
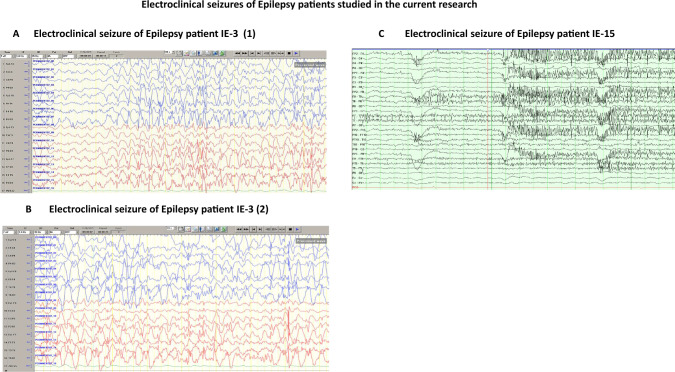
Electroclinical seizures of the Epilepsy patients IE-3 and IE-15 studied the current research. **A** Electroclinical seizure of the Epilepsy patient IE-3 showing onset of a generalized seizure that involves both hemispheres. **B** Electroclinical seizure of Epilepsy patient IE-3 showing continuation of the seizure lasting for ~ 50 s, manifested by loss of consciousness and generalized tonic clonic movement. **C** Electroclinical seizure of Epilepsy patient IE-15 showing a focal electroclinical seizure that started in the right posterior head region

In addition, representative electroclinical seizures of the two Epilepsy patients IE-3 and IE-15 are shown in Fig. 1A–C. For the Epilepsy patient IE-3, Fig. 1A shows the onset of a generalized seizure, that involves both hemispheres, and Fig. 1B shows a continuation of the seizure for ~ 50 s, manifested by loss of consciousness and generalized tonic–clonic movement. For the Epilepsy patient IE-15, Fig. 1C shows a focal electroclinical seizure that started in the right posterior head region. For controls, we tested in all the experiments several young healthy subjects.

### The two intractable enigmatic Epilepsy patients investigated in this study and a few others, have elevated levels of IgG antibodies in serum

First, we tested if the two intractable Epilepsy patients IE-3 and IE-15 have elevated levels of IgG in their serum. In the same tests we also measured the IgG levels of 13 other young intractable Epilepsy patients with somewhat enigmatic Epilepsy (all treated by Prof. H.G. Head of Epilepsy Dept., second author of this paper), and of nine control healthy subjects. We tested the IgG antibody levels because IgG antibodies represent approximately 75% of serum antibodies in humans, and are the most common type of antibody in blood circulation and extracellular fluids. High IgG level antibodies suggest possible ongoing humoral immunity, usually indicating a condition of an essential and beneficial immune response to a current or recent infectious organism. In contrast, autoimmune IgG antibodies are often pathogenic, and common among individuals with few autoimmune diseases: systemic lupus erythematosus, rheumatoid arthritis, and others.

**Fig. 2:**
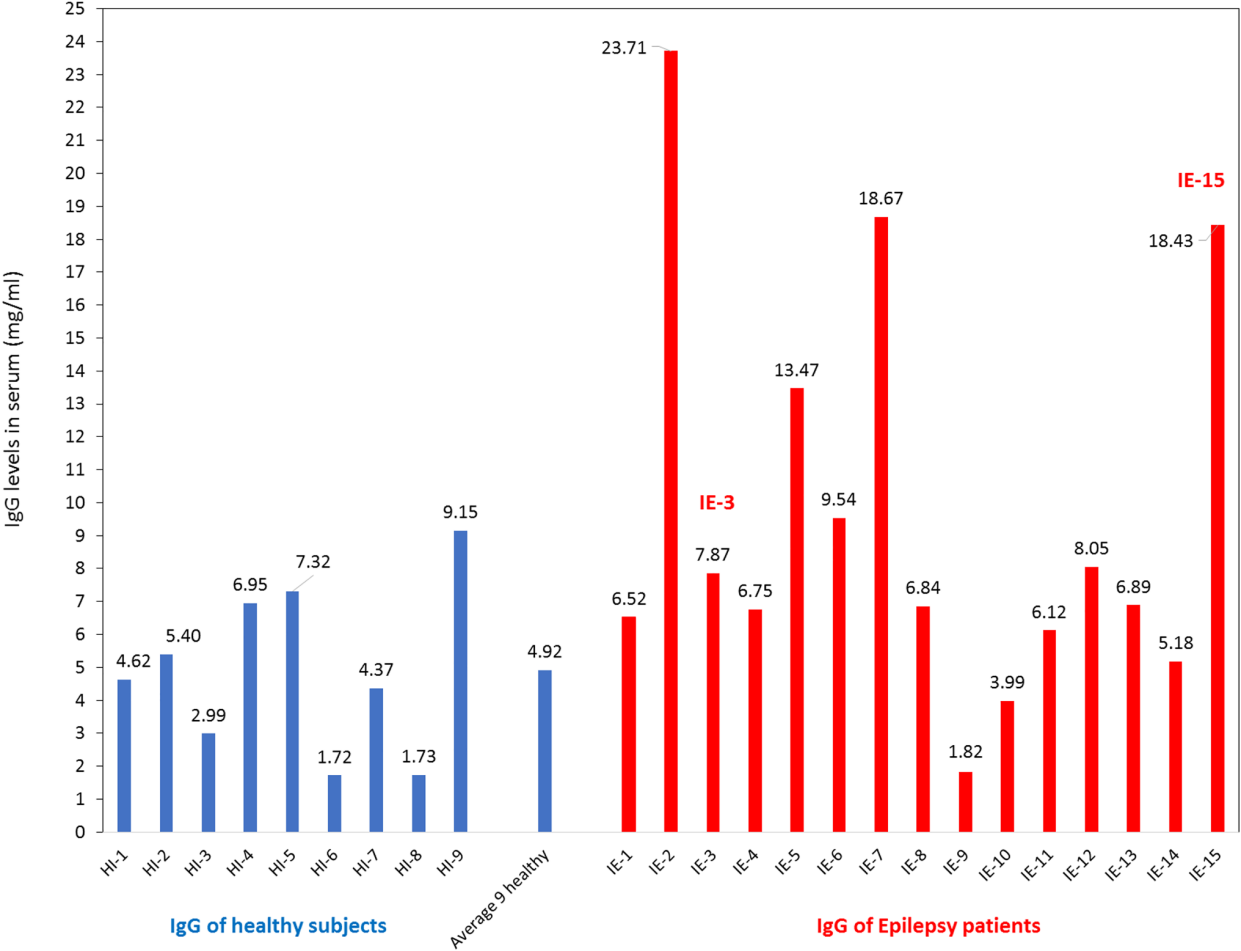
Some patients with severe intractable Epilepsy have a high level of IgG antibodies in their blood. The figure shows the concentration (mg/ml) of IgG antibodies in the serum of young patients with severe intractable Epilepsy including IE-3 and IE-15, and of control healthy subjects. The IgG antibodies were purified from serum samples of all the subjects in a professional protein laboratory within the Hebrew University (It should be noted that the IgG levels were tested only once for each Epilepsy patient or healthy subject, in single serum sample. Thus, statistical values could not be calculated

The evaluation of the IgG levels was performed for us in a professional protein laboratory, at the Wolfson Center of Applied Structural Biology, in the Hebrew University, Jerusalem, Israel. We found that the average ± SD IgG level in the serum of the 9 healthy subjects was 4.92 ± 2.42 mg/mL, and compared to that, the Epilepsy patients IE-3 and IE-15, and 5 additional young intractable Epilepsy patients: IE-2, IE-5, IE-6, IE-7, and IE-12—together 7 patients, have elevated levels of IgG antibodies in serum (Fig. 2).

The IgG level of IE-3 was 7.87 mg/ml, and that of IE-15 was 18.43 mg/mL (60% and 170% higher than the healthy controls, respectively, Fig. 2).

### The two intractable Epilepsy patients investigated in this study have elevated levels of antibodies to three different extracellular peptides of Ionotropic glutamate receptors

We designed and used a specific ELISA for detecting autoimmune antibodies in the serum of IE-3 and IE-15 that bind either of three relatively short extracellular antigenic peptides of ionotropic Glutamate receptors (iGluR), namely: AMPA GluR3**B** peptide, NMDA NR1 peptide, and NMDA NR2 peptide. Autoimmune antibodies to each of these Glutamate receptor peptides can bind neural cells and cause neural damage In vitro and In vivo, as shown in studies by several groups including ours, and discussed in previous publications (Levite 2002, 2014; Levite et al. 1999; Levite and Goldberg 2021; Cohen-Kashi Malina et al. 2006; Levite and Ganor 2008; Ganor et al. 2014, 2005c).

**Fig. 3:**
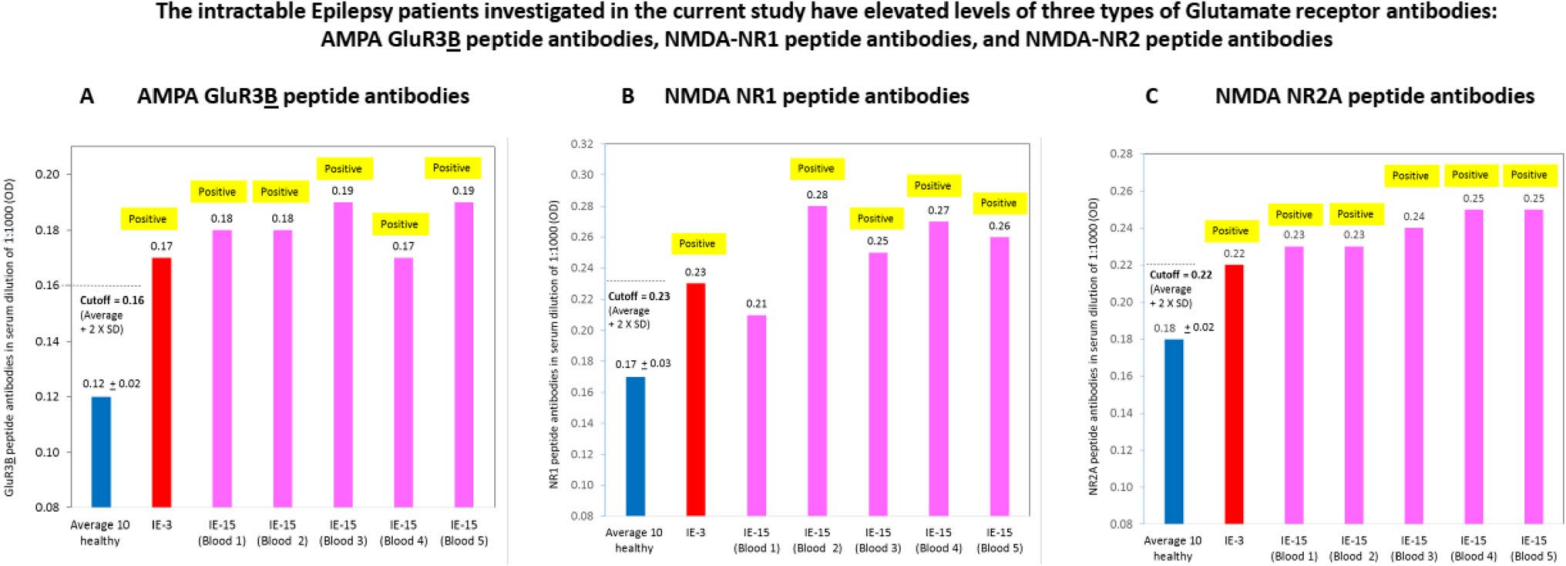
The intractable Epilepsy patients studied in the present research IE-3 and IE-15, were found to have elevated levels of three types Glutamate receptor antibodies in their serum: GluR3**B** peptide antibodies (**A**), NR1 peptide antibodies (**B**), and NR2A peptide antibodies (**C**), as compared to ten healthy subjects. Each of the above figures shows the average level of the respective type of Glutamate Receptor antibodies, in serum of 10 healthy subjects (blue bars), IE-3 (red bars), and 5 serum samples of IE-15 (derived from 5 blood withdrawals of IE-3) at different time points, pink). Each serum was tested in the ELISA in duplicate wells, in 3 different serum dilutions (1:10, 1:100 and 1:1000) for its parallel binding to either the respective GluR peptide, or to control PBS + 1% BSA. The figures show the level of the Glutamate receptor antibodies in very low serum dilution of 1:1000. The Y axis shows the value (in OD), calculated for each individual according to the following equation: specific binding (OD) of each individual serum, in each serum dilution, to the GluR peptide = Specific binding (OD) of this serum, in this serum dilution, to either AMPA GluR3**B** peptide (**A**) or NMDA-NR1 (**B**) or NMDA NR2 peptide (**C**) − (minus) the non-specific binding (OD) of this serum, in this serum dilution, to negative control PBS + 1% BSA (OD). The experimental cutoff seen in the figure shows the following value: average OD + (2 × SD) of the specific binding to the respective GluR peptide, of each serum of either the 7 healthy subjects tested in the same dilution (1:1000) in the same experiment

We found that IE-3 and IE-15 indeed have elevated levels of all three types of GluR antibodies in their serum, namely: AMPA GluR3**B** peptide antibodies (Fig. 3A), NMDA-NR1 peptide antibodies (Fig. 3B), and NMDA-NR2 antibodies (Fig. 3C), compared to ten healthy subjects. These specific GluR antibodies were detected at a very low serum concentration, i.e. very high serum dilution of 1:1000.

The results of one representative ELISA (Fig. 3A–C), show that a single blood sample of IE-3, and several blood samples of IE-15 taken at different time points, were all positive for GluR3**B** peptide antibodies, NR1 peptide antibodies, and NR2A peptide antibodies. This was evident by the fact that the patient’s OD readout of the GluR-specific ELISAs was above average + 2 × SD of the 10 healthy subjects.

### The two intractable Epilepsy patients investigated in this study do not have many other autoimmune antibodies found in various autoimmune diseases

Next, we tested if the IE-3 and IE-15 patients have various other autoimmune antibodies (listed below) in their sera, that are often present in some neurological autoimmune diseases. Since we could not perform these diagnostic tests in our own labs, the sera and CSFs of both patients were sent to an overseas professional diagnostic clinic: Euroimmun, Germany, that routinely performs these diagnostic tests.

The results obtained in Euroimmun are summarized in Table 3, and show that the blood and CSF of IE-3, which was tested twice at different time points, and of IE-15 tested once, are negative for all the tested neuronal autoimmune antibodies, directed against the following self-antigens: CASPR2, LGI1, GABA-RB1 Amphiphysin, CV2, PNMA1 (Ma2/Ta), Ri, Yo, Hu, Recoverin, Soxi, and Titin. These results strengthened the conclusion that the two Epilepsy patients IE-3 and IE-15 do not have a general non-specific autoimmunity characterized by many types of autoimmune antibodies, and that the Glutamate receptor antibodies we found in their sera (Fig. 3A–C) seem to be associated specifically with their Epilepsy.

**Table 3:** Diagnostic tests for autoimmune neuronal antibodies, done in external diagnostic clinics on the blood and CSF of the Epilepsy patients IE-3 and IE-15 studied in the current research

Test no.	Blood and CSF tested for neuronal autoimmune antibodies	Results
A. Diagnostic tests for neuronal autoimmune antibodies in the blood and CSF of the Epilepsy patient IE-3		
1	Anti-GABA-RB1 Abs, Anti-CASPR2 Abs, Anti-LGI1 Abs, Anti-NMDA-R Abs, Anti-AMPA-R1/2 (Glu1/2) Abs,	All negative
2	Anti-LGI1 Abs, Anti-GABA-B Abs, Anti-Amphiphysin Abs, Anti-CV2 Abs, Anti-PNMA1 (Ma2/Ta) Abs, Anti-Ri Abs, Anti-Yo Abs, Anti-Hu Abs, Anti-Recoverin Abs, Anti-Soxi Abs, Anti-Titin Abs, Anti-NMDR-R Abs, Anti-AMPA-R1/2 Abs	All negative

Test no.	Blood and CSF of tested for neuronal autoimmune antibodies	Results
B. Diagnostic tests for neuronal autoimmune antibodies in the blood and CSF of the Epilepsy patient IE-15		
1	Anti-GABA-RB1 Abs, Anti-CASPR2 Abs, Anti-LGI1 Abs, Anti-NMDA-R Abs, Anti-AMPA-R1/2 (Glu1/2) Abs	All negative

### The human neural cell culture used in the study contained both neurons and astrocytes

**Fig. 4:**
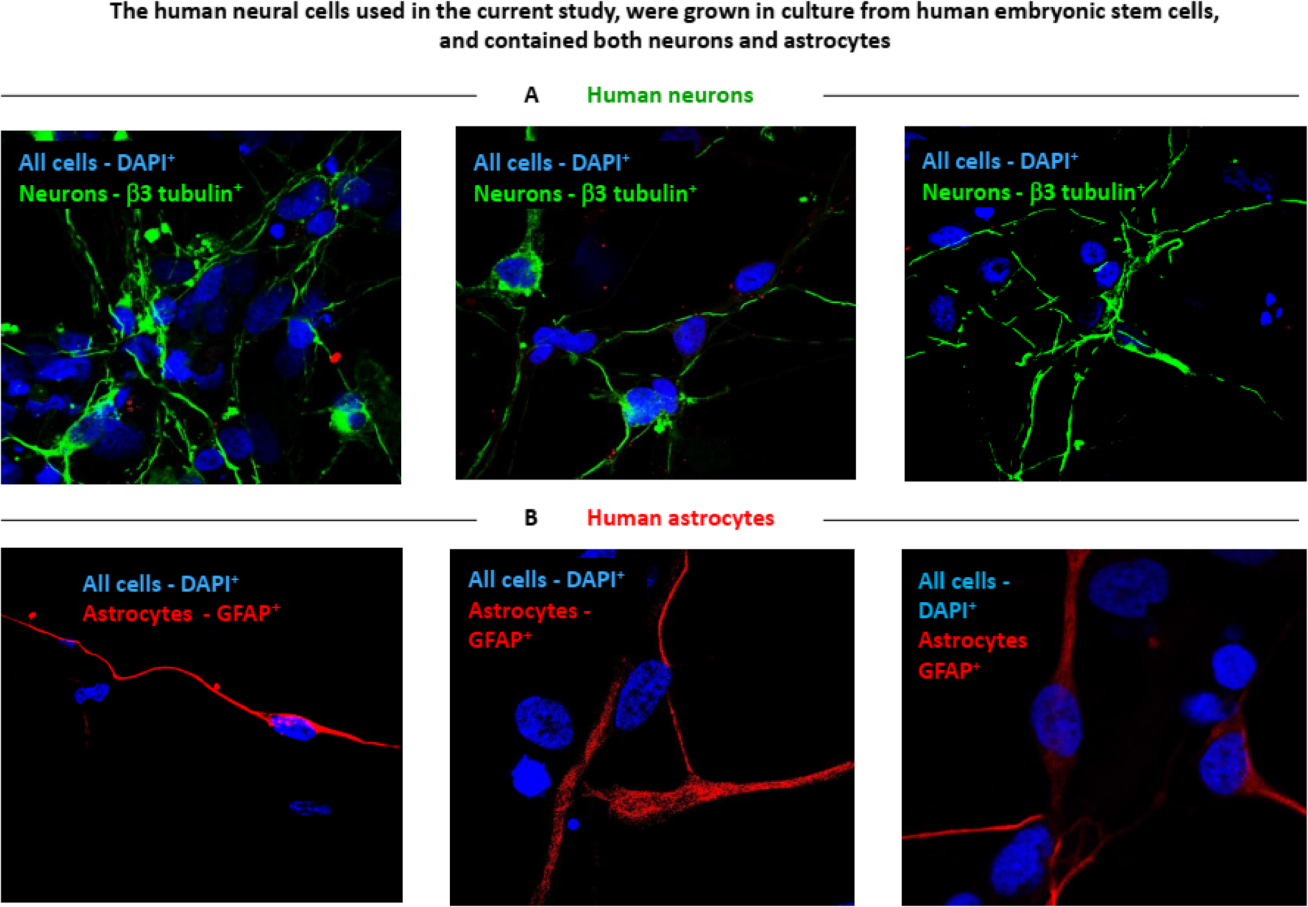
The human neural cells culture used in the current study contained both neurons and astrocytes. The human neural cells used in this study in several experiments, to test the binding and killing of the cells by the IgG of the Epilepsy patients, were grown and differentiated from human embryonic stem cells (hESCs), as previously described (Levite et al. 2020; Itsykson et al. 2005; Surmacz et al. 2012), and used successfully in various previous studies. The figure shows that this human neural cell culture contains both neurons (**A**) and astrocytes (**B**), demonstrated by specific immunostaining for each cell type: β3 tubulin for neurons, and GFAP for astrocytes, and representative confocal microscopy images. The three upper figures in part A of the figure show the β3 tubulin^+^ neurons stained in green, and all the DAPI^+^ cells stained in blue. The three lower figures in part **B** of the figure, show the GFAP^+^ astrocytes stained in red, and all the DAPI^+^ cells stained in blue

Next, we wished to test if the Epilepsy patient’s antibodies (regardless of their specificity) bind human neural cells. For this purpose, we grew and differentiated human neural cells from human embryonic stem cells (hESCs), as previously described and used successfully (Levite et al. 2020; Itsykson et al. 2005; Surmacz et al. 2012). Figure 4 shows that this human neural cell culture contains both neurons (Fig. 4A) and astrocytes (Fig. 4B). This was demonstrated by specific immunostaining of the human neural cells with either anti-β3 tubulin antibody for identifying neurons, or with anti-GFAP antibody for identifying astrocytes, and by representative confocal microscopy images showing the results of the immunostaining.

### IgG antibodies were affinity purified from the sera of the two intractable Epilepsy patients investigated in this study, for testing their effects In vitro and In vivo

For testing if the Epilepsy patient’s antibodies bind human neural cells, and for all the other In vitro and In vivo experiments planned in this study, we needed purified IgG antibodies of the Epilepsy patients and healthy control subjects. For this purpose, we sent the sera samples of IE-3 and IE-15 and healthy subjects for IgG purification, to a professional protein lab, at the Wolfson Center of Applied Structural Biology, at the Hebrew University, Jerusalem, Israel. There, the IgG of the patients and control subjects was purified by a routine widely accepted method of IgG purification. This method is described herein in ‘Materials and Methods’.

### Purified IgG of the two intractable Epilepsy patients investigated in this study bind human neural cells In vitro

**Fig. 5:**
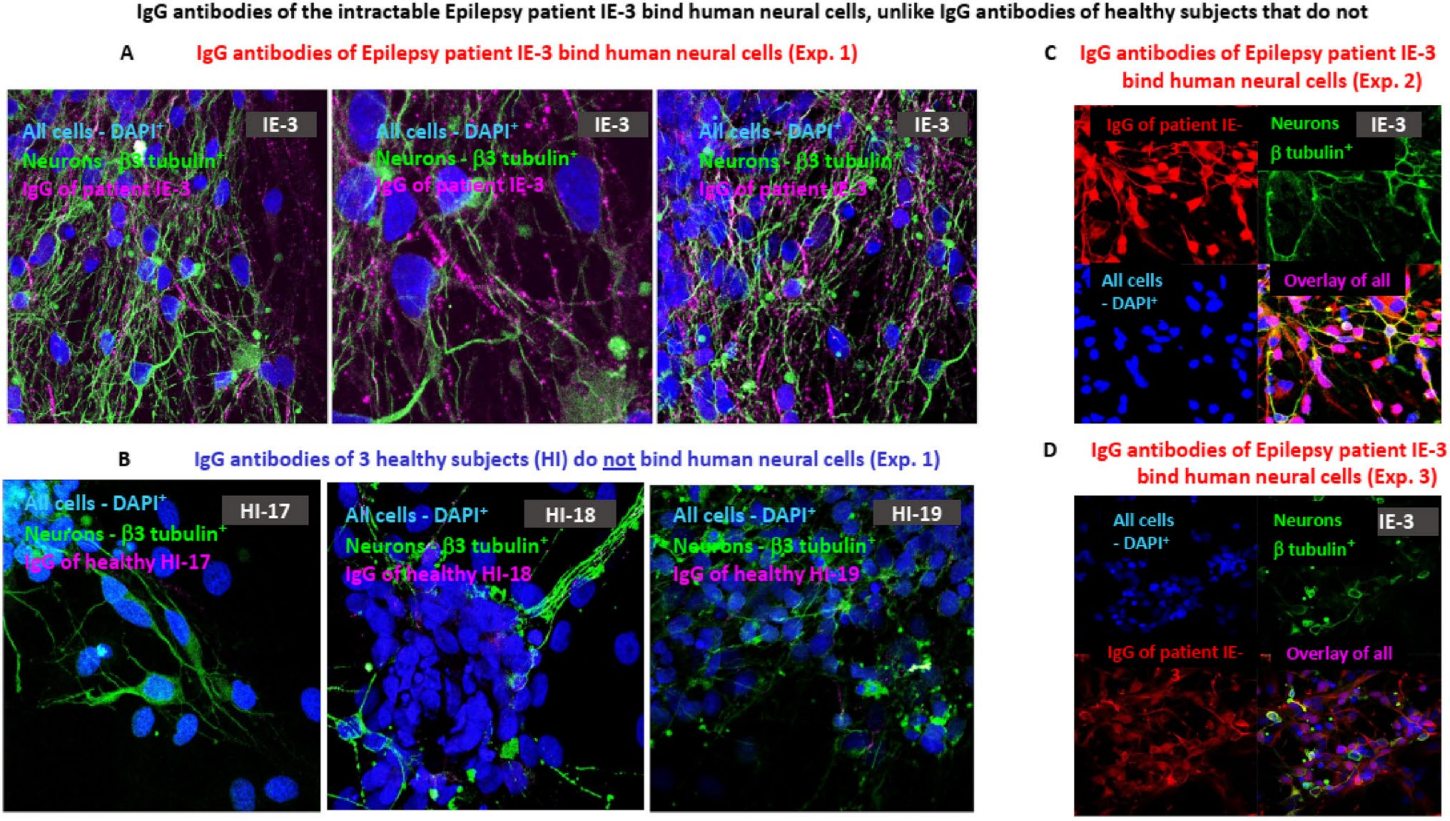
IgG antibodies of the intractable Epilepsy patient IE-3 bind human neural cells, unlike IgG antibodies of healthy subjects that do not. **A** Three representative confocal microscopy photos showing that purified IgG antibodies of the Epilepsy patient IE-3 bind human neural cells (pre-fixed before the binding assay). **B** Three representative confocal microscopy photos showing that purified IgG antibodies of three healthy subjects do not bind human neural cells. In A and B the neural cells bound by the human IgG are shown in pink, the β3 tubulin^**+**^ neurons are shown in green, and all the DAPI^**+**^ cells are shown in blue. **B**, **C** Similar to A, but of two additional separate binding experiments. The photos show the binding of purified IgG antibodies of the Epilepsy patient IE-3 to human neural cells. In these photos the neural cells bound by the human IgG are shown in red, the β3 tubulin^**+**^ neurons are shown in green, and all the DAPI^**+**^ cells are shown in blue

Next, we tested if the purified IgG antibodies of intractable Epilepsy patients investigated in this study can bind the human neural cells we grew from hESCs. In the first set of experiments, we tested only the IgG antibodies of the Epilepsy patient IE-3, and found, in three independent experiments, that the purified IgG antibodies of IE-3 indeed bind human neural cells (Fig. 5A, showing 3 representative photos taken from Exp. 1, Fig. 5C showing a representative photo from Exp.2, and Fig. 5D showing a representative photo from Exp. 3). In contrast, control purified IgG antibodies of healthy subjects did not bind human neural cells (Fig. 5B, showing single representative photos of each of three healthy subjects).

**Fig. 6:**
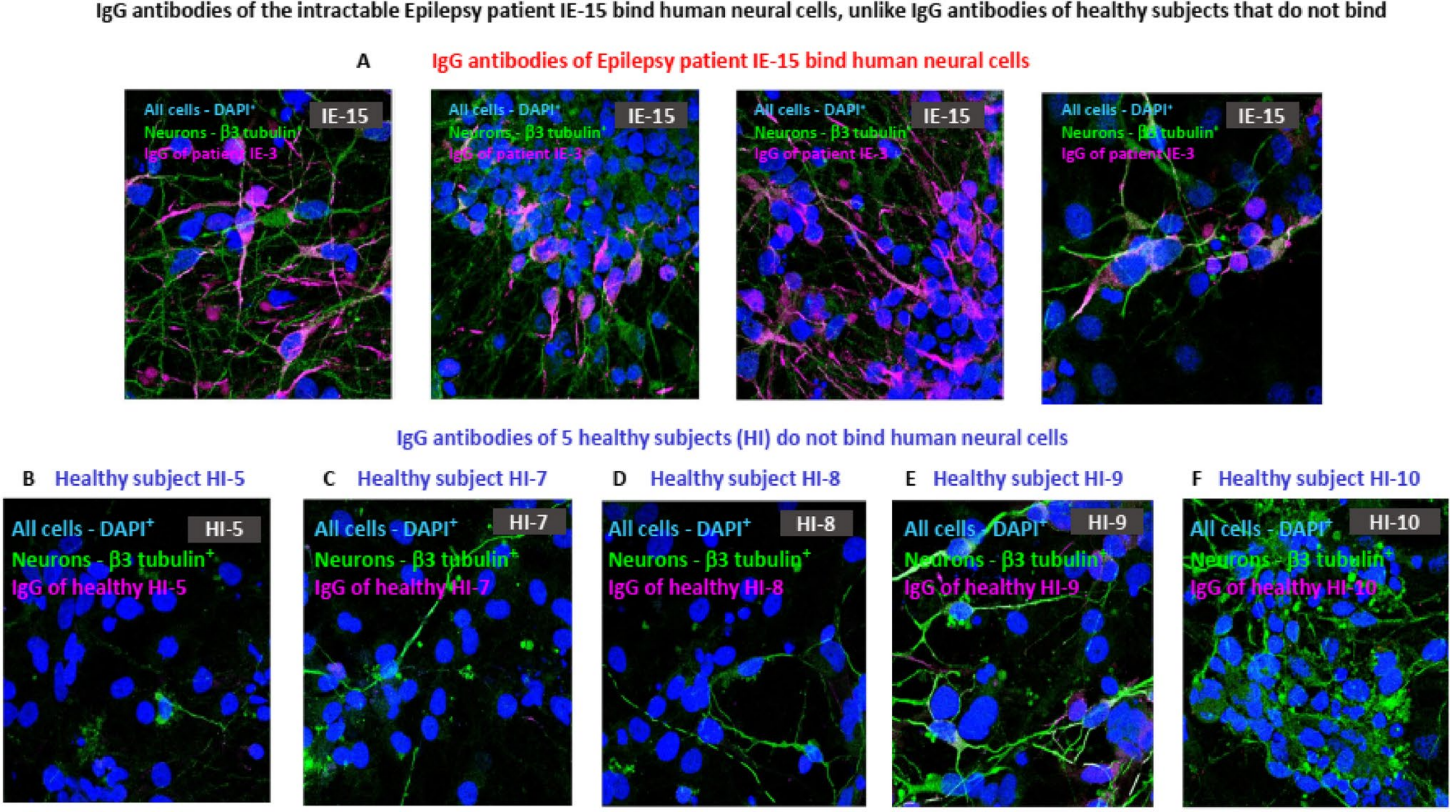
IgG antibodies of the intractable Epilepsy patient IE-15 bind human neural cells, unlike IgG antibodies of healthy subjects that do not. **A** Four representative confocal microscopy photos showing that purified IgG antibodies of the Epilepsy patient IE-15 bind human neural cells. **B**–**F** Five representative confocal microscopy photos showing that purified IgG antibodies of five different healthy subjects do not bind human neural cells

We further found that the purified IgG antibodies of the other Epilepsy patient-IE-15 also bind the human neural cells (Fig. 6A). In contrast, the IgG antibodies of five healthy subjects did not bind (Fig. 6B–F). Of note, the photographs shown in Fig. 6 are from another independent experiment that tested both the binding and the killing of the human neural cells by the IgG antibodies of IE-15 (described in the legend to Fig. 6, and in the next section of the ‘Results’).

### Purified IgG of the two intractable Epilepsy patients investigated in this study kill human neural cells In vitro

In the next set of In vitro experiments, we tested if the purified IgG antibodies of IE-3 and IE-15 patients can bind and then kill live (not fixed) human neural cells. In these experiments, the live cells were incubated with the IgG antibodies of the Epilepsy patients or healthy subjects, and then immunostained for their live vs. dead condition, and for IgG binding (as described in the respective ‘Method’ included in the ‘Supplemantary material’). The distinction of live and dead cells was performed by immunostaining the cells with SYTOX Green stain which is a dead-cell indicator. SYTOX Green is a high-affinity nucleic acid green-fluorescent nuclear and chromosome stain that does not cross the membranes of live cells, but easily penetrates cells with compromised plasma membranes.

**Fig. 7:**
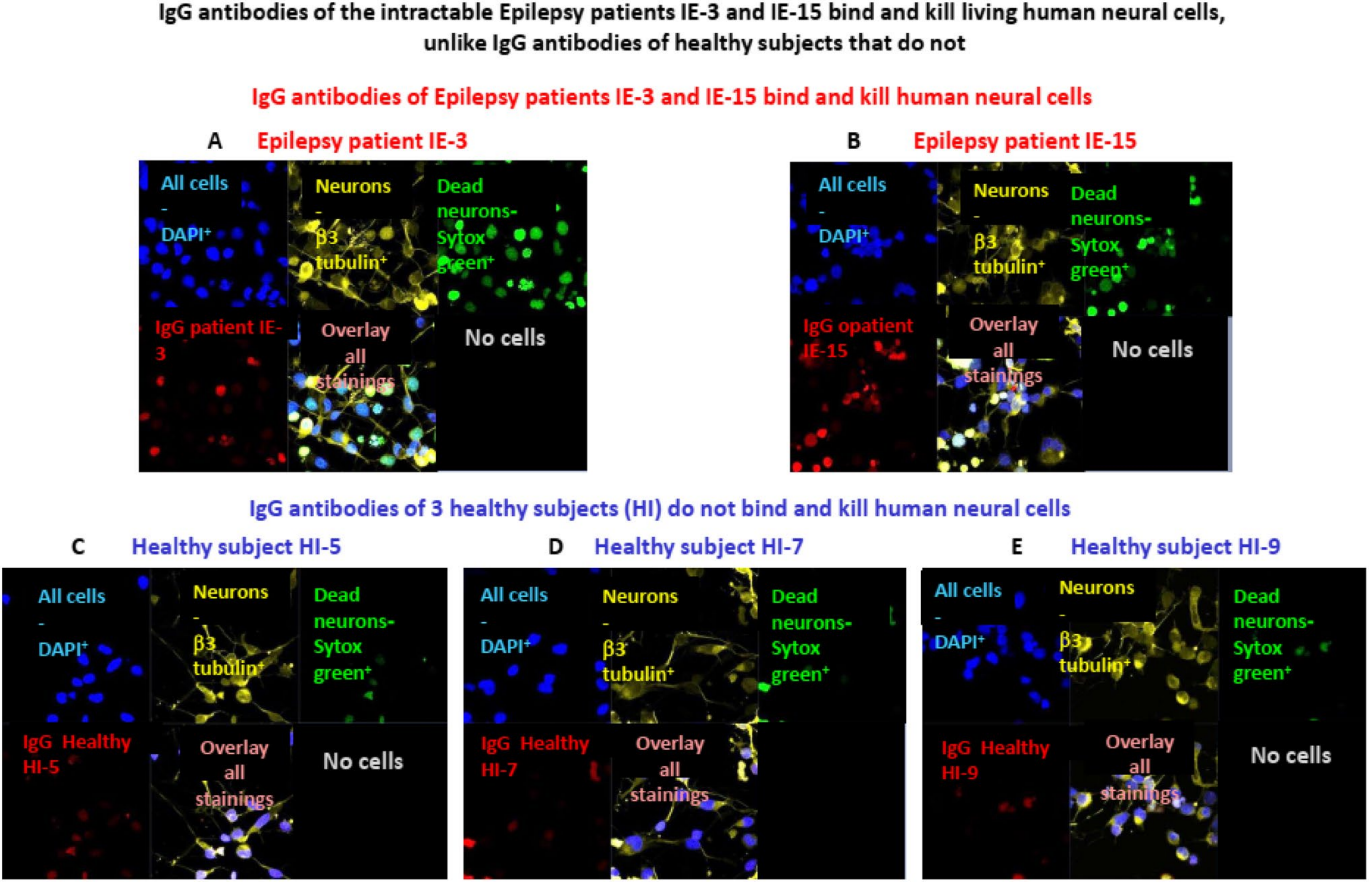
IgG antibodies of the intractable Epilepsy patients IE-3 and IE-15 bind and kill living human neural cells, unlike IgG antibodies of healthy subjects that do not. Representative confocal microscopy images show that purified IgG of the Epilepsy patients IE-3 (**A**) and IE-15 (**B**) bind (red staining) and kill (Sytox green staining) living (i.e. not pre-fixed, to allow binding and induction of functional effects that may led to cell death) human neural cells, within 1 h. while IgG of healthy subjects HI-5 (**C**) and HI-7 (**D**) and IH-9 (**E**) do not. In the big figures **A**–**E**, all the DAPI^+^ cells are shown in blue in the upper left internal small figures; the β3 tubulin^+^ neurons are shown in yellow in the upper middle figures, the Sytox green^+^ dead/necrotic neural cells are shown in green in the upper right figures, and the bound IgG is shown in red in the lower left figures. The lower middle figures show an overlay of all the stained components. The quantitative analysis of the bound and dead neural cells documented in these experiments is shown in Fig. 8

In these experiments, we found that the purified IgG antibodies of both IE-3 and IE-15 bind live human neural cells (see the Cy3 red-colored neural cells in Fig. 7A and B), and also kill these cells within 1 h only (see the neural cells colored with Sytox green in Fig. 7A, B respectively). In contrast, the IgG antibodies of control healthy subjects did not bind and kill these cells (see Fig. 7C–E, showing the results of five healthy subjects, all found to be negative for both binding and killing).

**Fig. 8:**
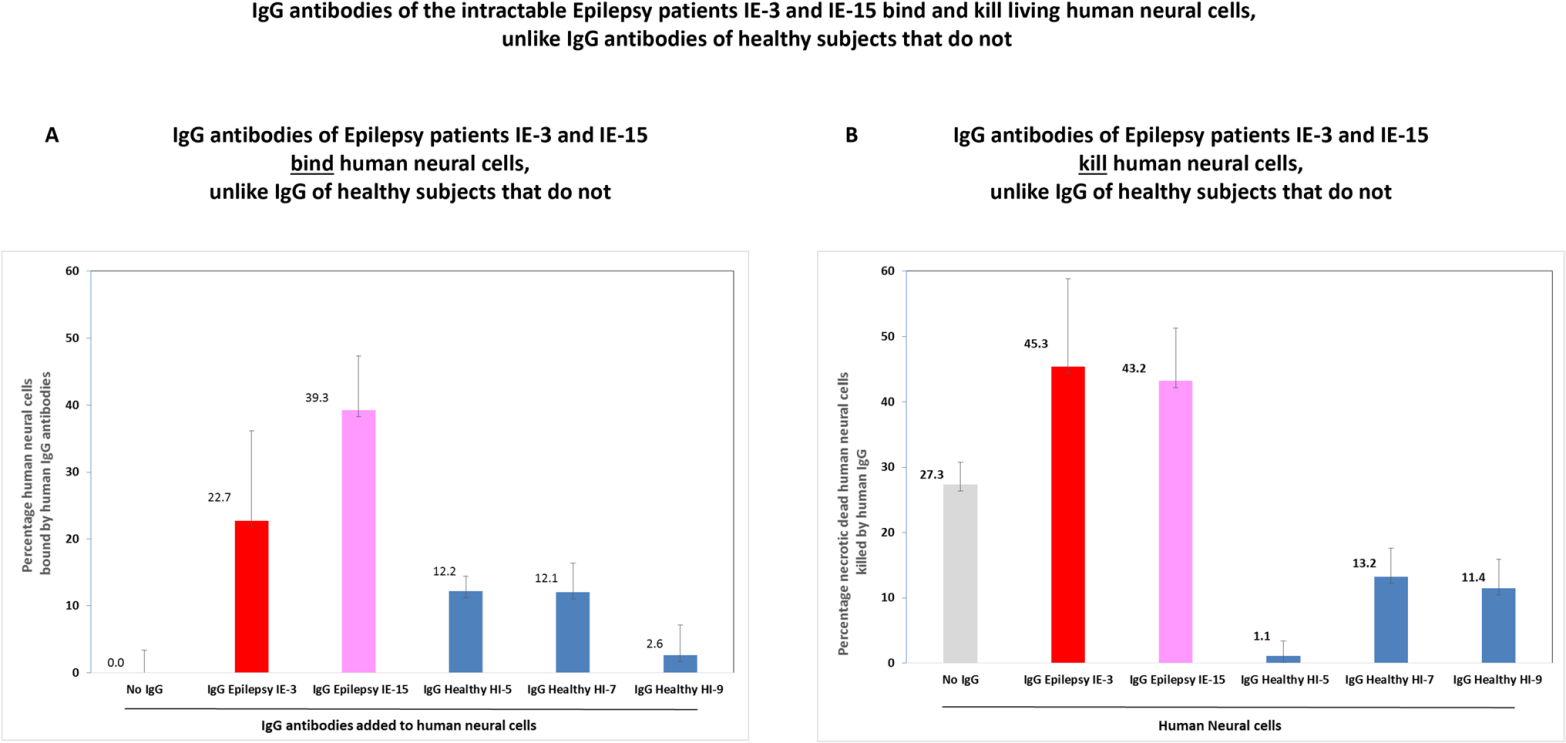
IgG antibodies of the intractable Epilepsy patients IE-3 and IE-15 bind and kill living human neural cells, unlike IgG antibodies of healthy subjects that do not. **A** Quantitative analysis of the percentage of human neural cells bound by purified IgG antibodies of the Epilepsy patients IE-3 or IE-15, or of three control healthy subjects. The quantitative analysis was performed based on confocal microscopy photos of the respective immunostainings. Two separate counts were made in each field of each confocal microscopy photographed image: first, all the DAPI^+^ cells were counted (seen in blue in Fig. 7), and secondly, only the cells to which the IgG antibodies bound were counted (seen in red or pink in Fig. 7). According to these numeric values, the percentage of human neural cells bound by the IgG antibodies of the Epilepsy patients or healthy subjects was calculated. **B** Quantitative analysis of the percentage of human neural cells killed by purified IgG antibodies of the Epilepsy patients IE-3 or IE-15, or of three control healthy subjects. The quantitative analysis was performed based on confocal microscopy photos of the respective immunostainings. Two separate counts were made in each field of each confocal microscopy photographed image: first, all the DAPI^+^ cells were counted (seen in blue in Fig. 7), and secondly, only the dead cells that stained by Sytox Green (seen in green in Fig. 7) were counted. According to these numeric values, the percentage of human neural cells killed by the IgG antibodies of the Epilepsy patients or healthy subjects was calculated

The quantitative analysis of these experiments, and the average percentage of human neural cells bound and killed by the the purified IgG antibodies of IE-3 and IE-15, in comparison to the negative binding and killing of the neural cells by the healthy subject’s IgG antibodies, is seen in Fig. 8A (binding) and Fig. 8B (killing).

The IgG antibodies of IE-3 bound on average 22.7 ± 8.7%, and those of IE-15 bound 39.3 ± 7.5%, of the neural cells. In comparison, the IgG antibodies of the three healthy subjects bound only 12.2 ± 19.4%, 12.1 ± 4.3%, and 2.6 ± 2.2% of the neural cells, respectively.

The analysis of the dead neural cells showed that in the untreated human neural cells (without addition of any IgG antibodies) there were 27.3 ± 3.4% dead cells. After adding the IgG antibodies of IE-3 there were 45.3% ± 13.5% dead human neural cells, meaning the IgG antibodies of IE-3 killed 45.3–27.3% = 18% of the cells. After adding IgG antibodies of IE-15 there were 43.2% dead human neural cells, meaning the IgG antibodies of IE-15 killed 16% of the cells. In contrast, after adding IgG antibodies of the three healthy subjects there were only: 1.1 ± 2.2%, 13.2 ± 4.3%, and 11.4 ± 4.5% dead human neural cells, respectively. Interestingly, comparison of the percentage of dead cells in the untreated human neural cells: 27.3 ± 3.4%, to that after the addition of the IgG antibodies of the healthy subjects: 1.1 ± 2.2%, 13.2 ± 4.3%, and 11.4 ± 4.5%, revealed an unexpected, interesting yet still unexplained phenomenon: the IgG antibodies of the healthy subjects had a beneficial rescuing effect on the human neural cells, resulting in a decrease in the % of dead cells.

### Establishing a new video EEG animal model in naïve rats for investigating human ‘Autoimmune Epilepsy’

**Fig. 9:**
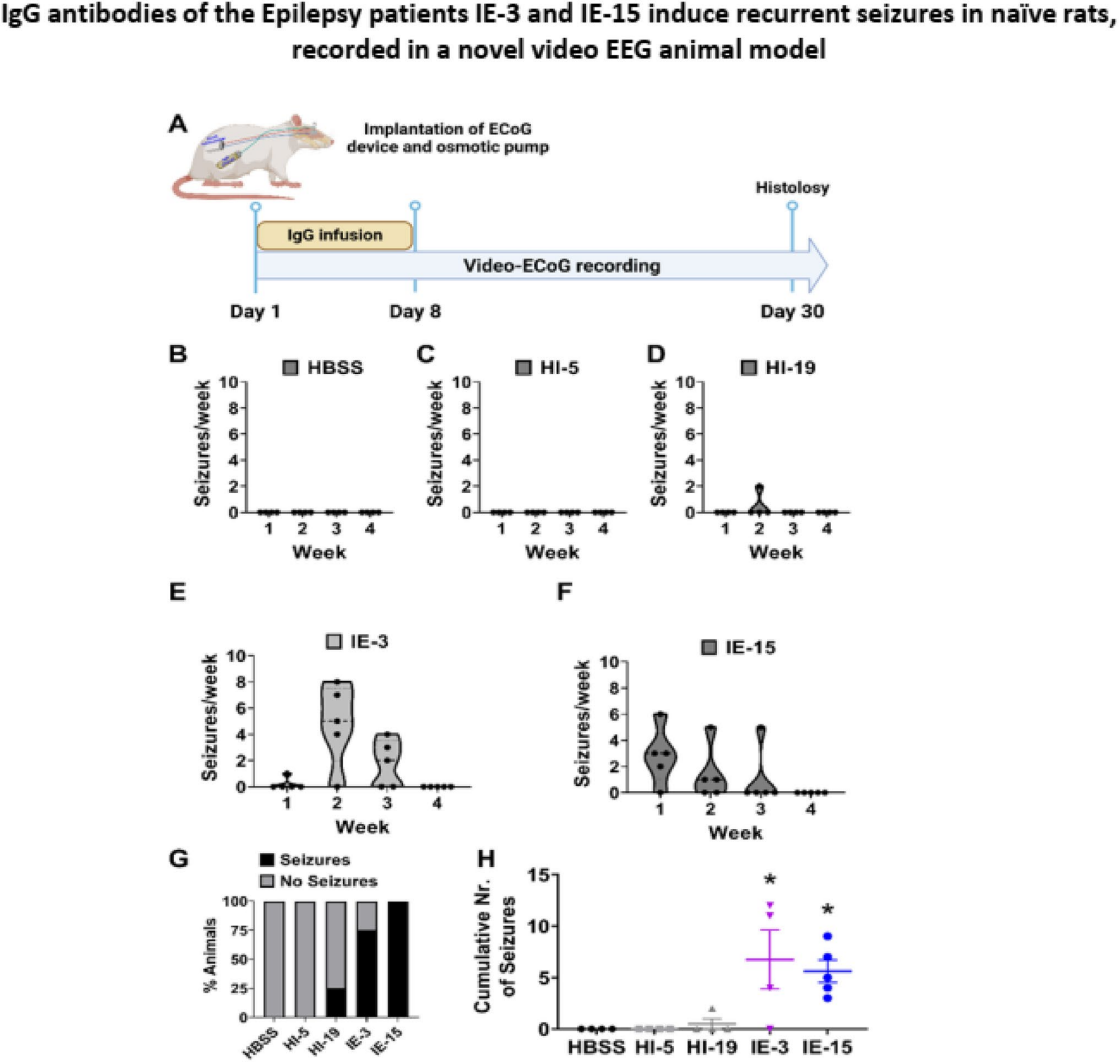
IgG antibodies of the Epilepsy patients IE-3 and IE-15 induce recurrent seizures in naïve rats, recorded in a novel video EEG animal model. **A** Schematic illustration of the experimental design. Seizure frequency per week in Sprague–Dawley rats infused with HBSS control (**B**), or purified IgG antibodies of healthy control subjects HI-5 and HI-19 (**C**, **D**, respectively), or purified IgG antibodies of the Epilepsy patients IE-3 or IE-15 (**E**, **F**, respectively). **G** Percentage of rats that developed seizures in the Epilepsy patients and their healthy controls. **H** Cumulative number of seizures in rats infused with HBSS, IgG antibodies of healthy control subjects HI-5 or HI-19, or the Epilepsy patients IE-3 or IE-15. Data analysis in **G** was performed using Chi-square and analysis in **H** was performed by one-way ANOVA followed by Dunnett's comparison test. Differences with *p < 0.05, **p < 0.01, ***p < 0.001 were considered statistically significant

We have established a new video EEG animal model in naïve rats for investigating whether purified autoimmune IgG of the intractable Epilepsy patients of IE-3 and IE-15-are able to bind neural cells in rat brain, damage the brain in specific brain regions, and induce recurrent GTCSs, if they are continuously present in the rat brain. The animal model and experimental design are illustrated schematically in Fig. 9A.

In brief, the preparation of the rats for this experiment consisted of several steps.

A subcutaneous ECoG transmitter with two subdural intracranial electrodes was implanted.

The recording electrode was placed above the right hippocampus (Paxinos and Watson 2014). The reference electrode was implanted in the contralateral hemisphere (Paxinos and Watson 2014). The electrodes were affixed to the skull. In addition, a brain infusion cannula was implanted into the right lateral ventricle of the brain (Paxinos and Watson 2014). The catheter tube was plugged for later connection to a mini osmotic pump, and a tunnel was created to position the tube into a subcutaneous cavity. Next, subcutaneously implanted osmotic minipumps were pre-loaded with purified IgG antibodies of either of the two intractable Epilepsy patients IE-3 or IE-15, or healthy control subjects. Then, the IgG antibodies were continuously infused from these minipumps into the rat’s brain for 1 week. The rats were monitored for seizure activity during that first week, and for additional 3 weeks, altogether for 4 weeks. The monitoring was done by wireless ECoG and video recording connected to a telemetry setup integrated with CCTV. The EEG-recorded data were analyzed for seizure activity and other EEG impairments. At the end of the experiment, the rats were sacrificed and perfused, and brain tissue samples were collected for further Immunohistochemical tests (as described in the following chapters of the ‘Results’).

### In vivo, video EEG studies revealed that IgG of the Epilepsy patients IE-3 and IE-15 induce seizures in naïve rats

We used our animal model described above to study the In vivo effects of purified IgG antibodies of the Epilepsy patients IE-3 and IE-15. We found that the IgG antibodies of these patients independently induced seizures in 75–100% of the tested animals (Fig. 9G).

 In contrast, IgG of healthy subjects did not induce seizures or did so to a lesser extent (0–25%, Fig. 9G). In all tested animals that received IgG antibodies of either of IE-3 or IE-15, higher numbers of seizures per week were recorded during weeks 1, 2, and 3 (Fig. 9E, F), as compared to the number of seizures induced by Hanks' Balanced Salt Solution (HBSS) control (Fig. 9B), and by the IgG antibodies of healthy subjects (Fig. 9C, D), that induced almost no seizure activity.

The cumulative number of seizures, in rats with seizures over the entire EEG monitored period, ranged between 5–15 and 2–10 for IE-3 and IE-15, respectively, while those of HBSS, HI-5, and HI-19 were 0, 0, and 2, respectively (Fig. 9H).

**Fig. 10:**
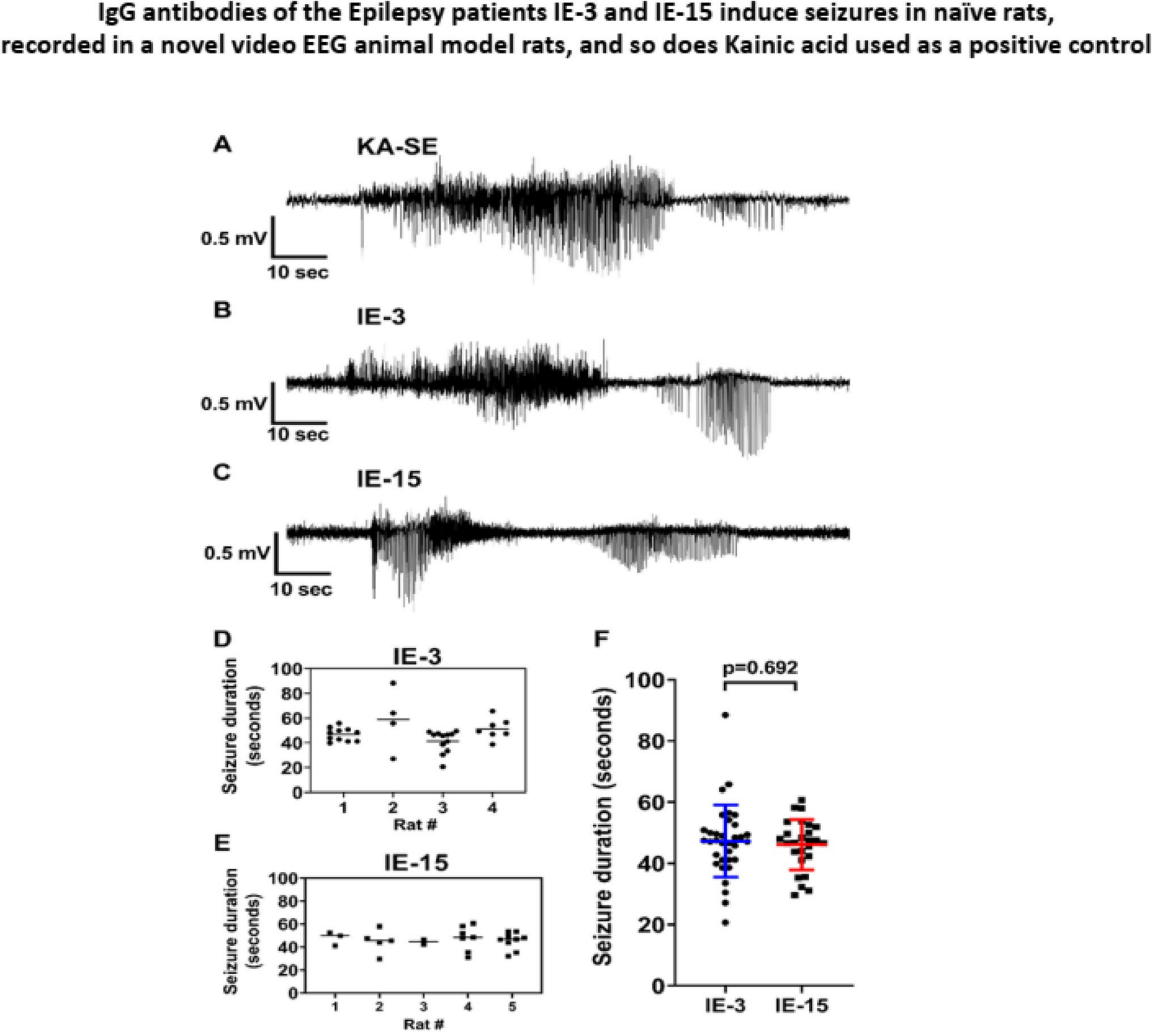
IgG antibodies of the Epilepsy patients IE-3 and IE-15 induce seizures in naïve rats, and so does Kainic acid used as positive control. **A** Representative sample traces of spontaneous seizures bursting in rat after status epilepticus induced by kainic acid. **B**, **C** Sample traces of the seizures induced in naïve rats by the IgG antibodies of the Epilepsy patients IE-3 and IE-15 respectively. **D**–**F** Duration of the seizures induced in naïve rats by the IgG antibodies of the Epilepsy patients IE-3 and IE-15 respectively**.** Statistical analysis in F was performed by unpaired T-test. Differences with *p < 0.05, **p < 0.01, ***p < 0.001 were considered statistically significant

We next found that seizure duration in rats that received IgG antibodies of the Epilepsy patient IE-3 lasted 20–90 s, and the seizure duration in rats receiving IgG antibodies of the Epilepsy patient IE-15 lasted 30–60 s (Fig. 10F).

The seizure traces in the rats that received IgG antibodies of IE-3 and IE-15 (Fig. 10B, C) showed an ictal EEG, starting with a high-frequency, low-amplitude activity that progressively evolved to high-amplitude regular polyspike, as compared to the Kainic acid-induced status epilepticus seizure traces (Fig. 10A) with high-amplitude rhythmic spike-waves (Tse et al. 2014).

### The IgG antibodies of the Epilepsy patients IE-3 and IE-15 bind and kill neuronal cells In vivo, in the CA3 brain region of the hippocampus of naïve rats

Our working assumption was that the binding and killing of neural cells in the rat’s brain by the IgG antibodies of the intractable Epilepsy patients IE-3 and IE-15 could have preceded the epileptic seizures that these antibodies caused, and contributed to their outburst.

**Fig. 11:**
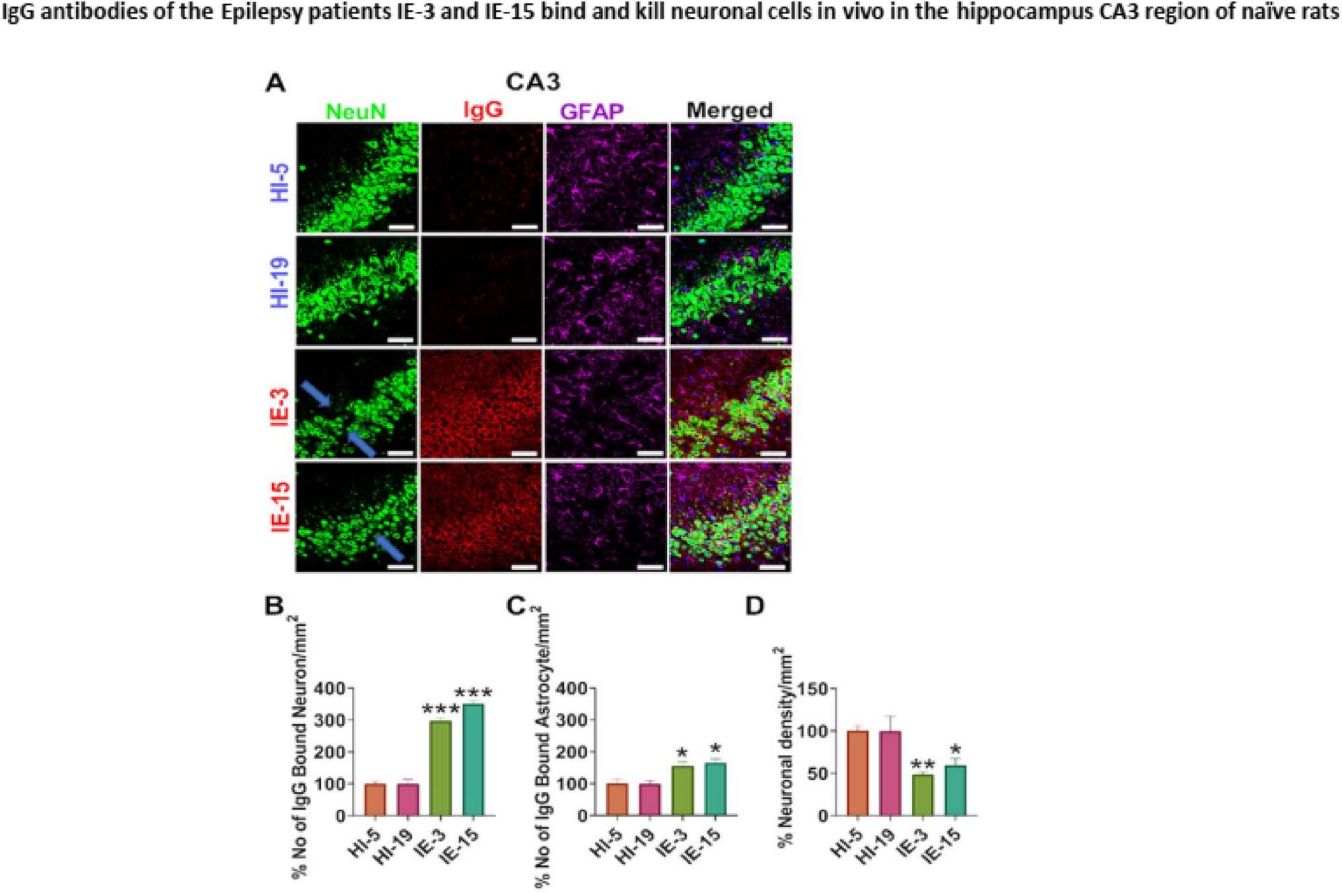
IgG antibodies of the Epilepsy patients IE-3 and IE-15 bind and kill neuronal cells in vivo in the hippocampus CA3 region of naïve rats. **A** Representative images of immunostaining of brain slices of the hippocampus of naïve rats post infusion of purified IgG antibodies either of healthy control subjects (HI) or of the two intractable Epilepsy patients (IE): IE-3 and IE-15. The pictures in the left column, entitled “NeuN” show staining of neuronal cells. The pictures in the second column to the left, entitled “GFAP” show staining of astrocytic cells. The pictures in the third column to the left, entitled “IgG” show staining of the human IgG (patient’s IgG) antibodies. The pictures in the right column, entitled “Merged” show an overlap of the staining. **B** Percentage of *neurons* in the CA3 brain region of the rat hippocampus bound by the human IgG of either the Epilepsy patients IE-3 or IE-15, or the healthy control subjects. **C** Percentage of *astrocytes* in the CA3 brain region of the rat hippocampus bound by the human IgG of either the Epilepsy patients IE-3 or IE-15, or the healthy control subjects. **D** Percentage neuronal density in the CA3 region of the rat hippocampus bound by the human IgG of either the Epilepsy patients IE-3 or IE-15, or the healthy control subjects. Scale 50 μm. Data are displayed as the mean ± SEM, *p < 0.05, **p < 0.01, ***p < 0.001 analyzed by one-way ANOVA followed by Dunnett's comparison test

Immunohistochemical analysis of the brains of the experimental rats, removed at the end of the EEG recording period (4 weeks), revealed that the IgG antibodies of IE-3 and IE-15 indeed bound and killed neuronal cells (Fig. 11A, B) and astrocytic cells (Fig. 11A, C) in the CA3 brain region of the rat hippocampus (Fig. 11). This was evident by a dramatic reduction in the number of neurons (Fig. 11D) at the CA3 brain region in the rats that were infused with the IgG antibodies of either IE-3 or IE-15. In contrast, the IgG antibodies of the healthy control subjects neither bound nor killed neural cells in the CA3 Hippocampal region, or did so to a significantly lesser extent (Fig. 11A, B).

Thus, the numbers of neurons to which the IgG of IE-3 and IE-15 bound in the CA3 region of the hippocampus were significantly higher than the number of neurons bound by the IgG antibodies of the control healthy subjects (Fig. 11B) [F = (3,12) = 153.4, p < 0.0001].

Moreover, the neuronal density was dramatically reduced (Fig. 11D) [F = (3,12) = 7.082, p = 0.0054] in rats that received Epilepsy patient’s IgG antibodies, as compared to the IgG antibodies of their healthy counterparts.

Furthermore, we found that the purified IgG antibodies of Epilepsy patients IE-3 and IE-15 destroyed the integrity of intact neural networks in the rat’s brain, as evident by the disappearance of intact immunofluorescent-labeled neurons (Fig. 11A, arrows), in contrast to the lack of similar effects induced by the IgG antibodies of the healthy control subjects.

### The IgG antibodies of the Epilepsy patients IE-3 and IE-15 bind and kill neuronal cells In vivo, in the cortex of naïve rat’s brains

**Fig. 12:**
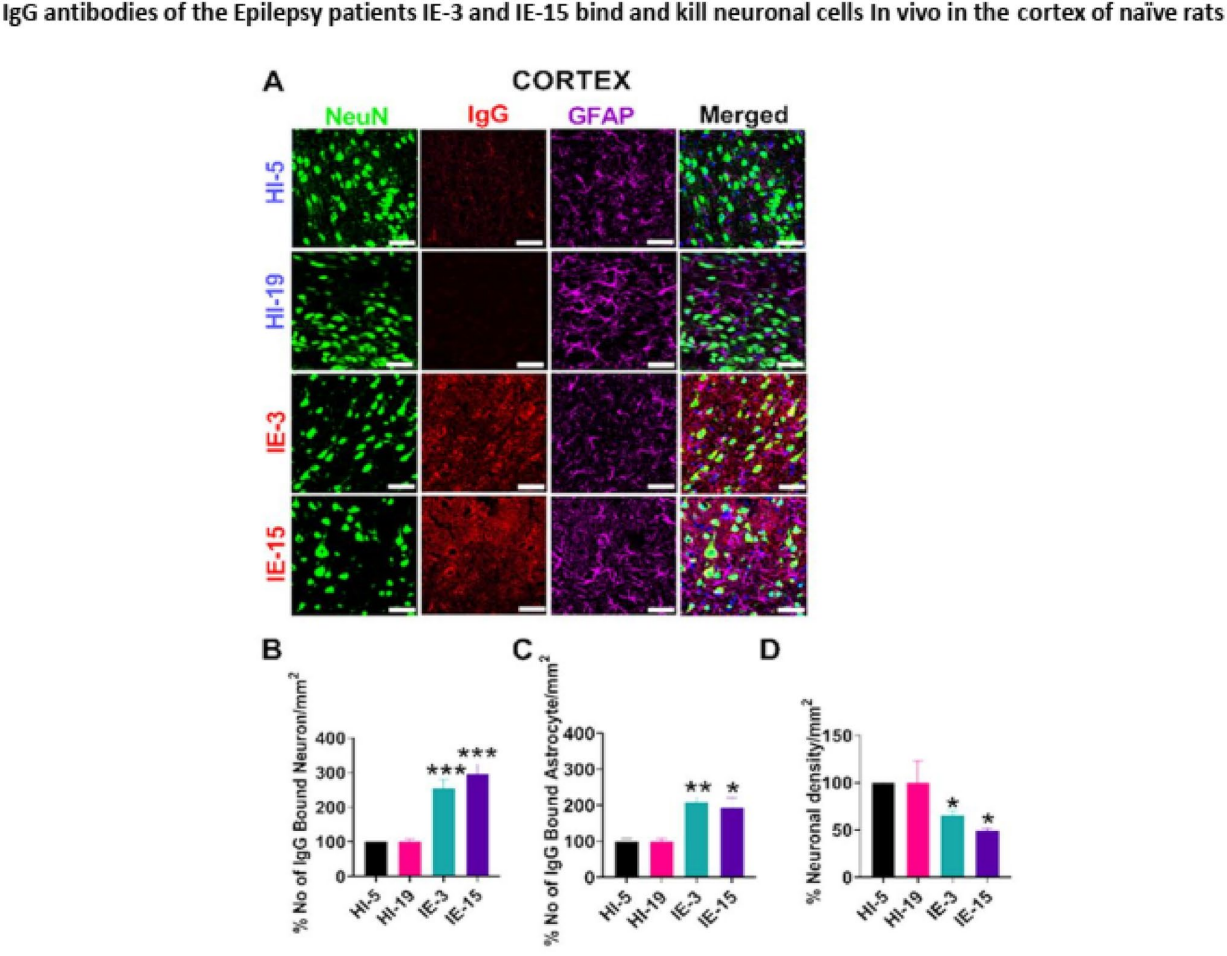
IgG antibodies of the Epilepsy patients IE-3 and IE-15 bind and kill neuronal cells In vivo in the cortex of naïve rats. **A** Representative images of immunostaining of brain slices of the cortex of naïve rats post-infusion of purified IgG antibodies either of healthy control subjects (HI) or of two intractable Epilepsy patients IE-3 and IE-15. The pictures in the left column, entitled “NeuN” show staining of neuronal cells. The pictures in the second column to the left, entitled “GFAP” show the staining of astrocytic cells. The pictures in the third column to the left, entitled “IgG” show staining of human IgG (patient’s IgG) antibodies. The pictures in the right column, entitled “Merged” show an overlap of all the staining. **B** Percentage number of neurons in the cortex of naïve rats bound by the human IgG of either the Epilepsy patients IE-3 or IE-15, or the healthy control subjects. **C** Percentage number of astrocytes in the cortex of naïve rats bound by the human IgG of either the Epilepsy patients IE-3 or IE-15, or the healthy control subjects. **D** Percentage neuronal density in the cortex of naïve rats bound by the human IgG of either the Epilepsy patients IE-3 or IE-15, or the healthy control subjects. Scale 50 μm. Data are displayed as the mean ± SEM, *p < 0.05, **p < 0.01, ***p < 0.001 analyzed by one-way ANOVA followed by Dunnett's comparison test

We further found that the IgG antibodies of the Epilepsy patients IE-3 and IE-15 bound and killed neuronal cells in the cortex of the rat’s brain (Fig. 12A). A significantly higher number of IgG-bound neurons (Fig. 12B) and astrocytes (Fig. 12C) in these brain regions were counted in rats that received IgG antibodies of IE-3 and IE-15, as compared to the IgG antibodies of the healthy control subjects [F = (3,12 = 27.05, p < 0.0001]. In line with that, we found a significantly lower number of neuronal cells in the rat cortex (Fig. 12D) [F = (3,12) = 4.500, p = 0.0246]. The statistical analysis of this set of data was done by one-way ANOVA followed by Dunnett's comparison test.

## Discussion

### The aim and main findings of the present study

**Fig. 13:**
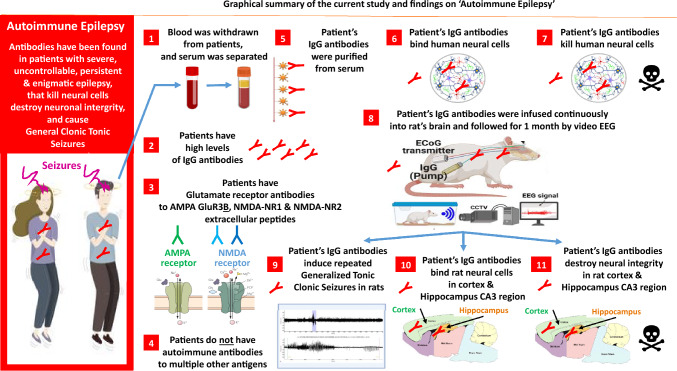
Graphical summary of the current study on ‘autoimmune Epilepsy’, and of all its findings

The current study was performed to try resolving the mystery of two intractable Epilepsy patients, and test for evidences of ‘Autoimmune Epilepsy’. These Epilepsy patients were enigmatic for many years before this study because of the negative results of numerous tests performed in the hospital prior to this study, specified in Table 2. Our study revealed several findings regarding the Epilepsy patients studied, which are listed in brief in this section, and schematically drawn in the Graphical Abstract (Fig. 13). Some of our discoveries and their conclusions, and citation of relevant studies, are discussed further in separate paragraphs, below the list of findings.

1st finding: The Epilepsy patients we studied have elevated IgG antibodies in blood.

2nd finding: The Epilepsy patients have elevated levels of three types of Glutamate receptors antibodies, namely: GluR3B peptide antibodies, NMDA-NR1 peptide antibodies, and NMDA-NR2 peptide antibodies. Previous studies have shown that each of these GluR antibodies can bind and damage neural cells in tissue culture, and cause brain damage In vivo [reviewed in Levite and Goldberg (2021)]. We elaborate on the pathogenic activity of these GluR antibodies in a separate section below.

3rd finding: The Epilepsy patient’s IgG antibodies bind human neural cells In vitro.

4th finding: The Epilepsy patient’s IgG antibodies kill human neural cells In vitro.

5th finding: The Epilepsy patient’s IgG antibodies induce repeated GTCs in naïve rats, following their continuous infusion into naïve rat’s brain ventricular region.

6th finding: The Epilepsy patient’s IgG antibodies bind rat neurons and astrocytes in the cortex and hippocampus CA3 region of naïve rats.

7th finding: The Epilepsy patient’s IgG antibodies induce neuronal loss in the cortex and hippocampus CA3 region of naïve rats.

### The patient’s IgG antibodies induced GTCs in naïve rats

We found that the type of seizures generated by the purified IgG antibodies of the Epilepsy patients IE-3 and IE-15 in naïve rats was typical of GTCs. This conclusion is based on the fact that the repeated seizures featured an Ictal EEG showing generalized polyspikes, building up to an increasing frequency in the tonic phase, followed by a generalized spike-wave pattern that gradually slows to a lower frequency (Smith 2005), and eventually a postictal suppression follows termination of the seizure.

We further found that the average duration of the seizures induced by the Epilepsy patient’s IgG antibodies was 68 s. The seizure duration ranged between 20 and 90 s, which is typical of the duration of GTCs that last up to 90 s or less, as described by Britton et al. (2016).

Wei et al. (2021) employed the use of EEG monitoring for the detection of spontaneous seizures in multiple experimental mouse models of Epilepsy. In that study, when the duration of the seizure event was greater than 10 s, it was defined as a seizure (Wei et al. 2021).

The type, duration and recurrent seizures induced by the Epilepsy patient’s IgG antibodies in naïve rats in the present study strongly suggest that the patient’s autoimmune IgG antibodies are inducing such seizures, i.e. recurrent GTCSs in the patient’s own body.

If this happens, these Epilepsy patients indeed suffer from ‘Autoimmune Epilepsy’.

EEG proves invaluable in assessing individuals suspected of seizures, Epilepsy or experiencing atypical episodes. In nearly all cases of Epilepsy, distinct EEG changes manifest during seizures (ictal recordings). Furthermore, many Epilepsy patients exhibit identifiable interictal epileptiform discharges (IEDs), such as spikes (lasting < 70 s), spike and wave, or sharp-wave discharges (lasting 70–200 s) (Paxinos and Watson 2014).

Our study and findings in naïve rats show that testing Epilepsy patients’ purified IgG antibodies (not serum!) In vivo, in similar animal models to the ones we developed, seems to be an essential and appropriate method to find out if a given Epilepsy patient has seizure-inducing autoimmune antibodies, and prove their pathogenicity, and as such to diagnose that the patient suffers from ‘Autoimmune Epilepsy’ and that he/she should be treated accordingly. The sophisticated video EEG tests we performed in naïve animals are expensive and complex and require profound unique knowledge, specific experience and appropriate very costly equipment. As such, they are not in hand in the majority of hospitals. Due to that, we recommend that whenever possible, small serum samples of patients with intractable enigmatic Epilepsy will be sent for In vitro and In vivo investigation, to external laboratories in the Academic Institutes or elsewhere, that have, or will be able to establish, animal models such as the video EEG setup we have established, and used successfully.

### Brain damage in the cortex and the hippocampus CA3 region, as caused in this study in rats by IgG antibodies of Epilepsy patients, can induce seizures and other neurological problems in the patients themselves

Paroxysmal alteration of neurological function caused by an excessive hypersynchronous neuronal discharge in the brain is known as seizure. The hippocampus, amygdala, frontal cortex, temporal cortex, and olfactory cortex are the common areas involved in seizures (Chauhan et al. 2022). Brain lesions are the most common cause of adult‐onset Epilepsy (Nordberg et al. 2023).

**The brain’s cortex.** The cerebral cortex, also called the gray matter, is the brain’s outermost layer of nerve cell tissue. The cerebral cortex carries out essential brain functions, including memory, thinking, learning, reasoning, problem-solving, emotions, consciousness, and sensory functions. In general, lesions in the cerebral cortex are associated with a higher risk for Epilepsy than lesions in other parts of the brain (Pitkanen et al. 2016; Kerkhof and Vecht 2013).

**The hippocampal CA3 brain region.** The hippocampus CA3 region is important for rapid encoding of memory (Rebola et al. 2017). In recent years, the CA3 region has attracted major attention not only for its central role in memory, but also for its specific role in susceptibility to seizures and neurodegeneration [see for example a paper entitled “The CA3 region of the hippocampus: how is it? What is it for? How does it do it?”(Cherubini and Miles 2015)]. Internal connectivity in the CA3 subfield is richer than in other hippocampal regions (Cherubini and Miles 2015). Recurrent axon collaterals of CA3 pyramidal cells ramify extensively, making excitatory contacts with neighboring excitatory and inhibitory neurons. Evidence obtained in an animal model shows that the CA3 excitatory output is required for both the generation of epileptiform oscillatory activity and the progression of behavioral seizures (Yu et al. 2016).

Taken together, multiple direct and indirect evidences described in reliable papers show that damage in the cortex and the hippocampus CA3 region can lead to, or at least contribute to, outbursts of seizures and multiple other impaired brain functions.

Based on these evidences, we conclude that autoimmune antibodies—Glutamate receptor antibodies and/or others—of Epilepsy patients, that damage neural cells in the cortex and hippocampus CA3 region, as the purified IgG of the Epilepsy patients IE-3 and IE-15 induced in naïve rats in the present study, can induce or contribute to the patient’s own seizures.

In addition, we suspect that Epilepsy patient’s autoimmune antibodies that damage the patient’s cortex and the hippocampus CA3 region could not only induce seizures, but also: impair multiple other brain functions, among them thinking, learning, reasoning, understanding, memorizing, solving problems and others, and cause abnormal behavior, emotions, consciousness, and sensory functions, and even lead to psychiatric problems.

### What can be studied in animal models cannot be studied in the patients themselves

A question that could *not* be answered in this study is whether the Epilepsy patient’s IgG antibodies indeed induce seizures and brain damage in the patient’s own body, like they did in naïve rats in the current study. This critical question will be left without a decisive and unequivocal answer, and only with a reasonable assumption that this is indeed the case, because it is impossible to carry out suitable In vivo research on the brain of a living Epilepsy patient, and isolate the seizures, brain damage and abnormal neural functions caused solely by the patient’s own autoimmune antibodies. Therefore, the development and usage of reliable and applicable animal models that make it possible to imitate what happens in the patients themselves, is so important.

### The most important information for the Epilepsy patients themselves is whether they have pathological autoimmune antibodies, regardless of their antigenic specificity

The specific antigenic identity of the autoimmune antibodies of the Epilepsy patients IE-3 and IE-15 that induced all the pathological effects documented in this study by the patient’s IgG antibodies In vitro and In vivo was *not* determined in this study, since we studied and observed all the functional pathological effects, both in tissue culture and in the rats, with a purified preparation of all the IgG antibodies present in the patient’s sera.

Purified biological material that contains all the antibodies of IgG class surely contains IgG antibodies directed against many different non-self and self-antigens. That being said, let us argue that while testing a heterogeneous population of patient’s IgG antibodies, rather than only one type of autoimmune antibody to a known antigen, can be considered a scientific disadvantage, the most important information for the patients themselves, and to the clinicians that treat them, is whether or not the patient has *any type* of pathological autoimmune antibodies that induce seizures, neural death and impaired brain function. Their antigenic identity of the autoimmune antibodies is of value of course but of secondary importance, since available immunotherapies for most if not all autoimmune diseases are *not* tailored to autoimmune antibodies with a distinct antigenic specificity. Rather, they either silence, neutralize or purge many types of antibodies, with different specificities.

### Glutamate receptor antibodies in intractable Epilepsy and Nodding syndrome

We suspect (but cannot prove) that in the case of the Epilepsy patients IE-3 and IE-15, it is their own Glutamate Receptor antibodies that bind neural cells, damage the brain and induce GTCSs. This suspicion is based on: (a) our finding that these Epilepsy patients have 3 types of GluR antibodies in their sera, and (b) these specific types of GluR antibodies, have already been shown to induce multiple pathological effects In vitro and In vivo [reviewed in Levite and Goldberg (2021) and summarized also in the next paragraphs].

Regretfully, we could not affinity-purify sufficient amounts of each type of the patient’s Glutamate rector antibodies from their own small blood samples, to perform all the functional studies also with them, and compare their effects to those induced by their total IgG antibodies, and by control antibodies of healthy subjects.

Various types of autoimmune antibodies to Glutamate receptors were identified in various diseases and shown to have pathological effects (Levite and Goldberg 2021; Levite 2014; Levite et al. 2020). Of all types of Glutamate receptor antibodies, the AMPA-GluR3**B** peptide antibodies seem to be the most exclusive to Epilepsy since they were found so far only in some patients with Epilepsy. Elevated levels of GluR3 antibodies, especially GluR3**B** peptide antibodies, were found so far in the serum of about 35% of > 370 persons with severe, intractable and enigmatic Epilepsy of various types (Rogers et al. 1994; Twyman et al. 1995; Andrews and McNamara 1996; He et al. 1998; Ganor et al. 2004; Levite 2014; Lai et al. 2022).

Recently, we also found elevated GluR3**B** peptide antibodies in 86% of young South Sudanese patients with the devastating and often fatal pediatric Epilepsy: Nodding syndrome (NS). Based on our own findings in this study (Levite et al. 2020) (summarized in the next section), and those of Johnson et al. (2017), we conclude that NS is most probably an ‘Autoimmune Epilepsy’. GluR3**B** peptide antibodies were also found in the CSF of some Epilepsy patients [see for example (Ganor et al. 2004)]. The GluR3**B** peptide antibodies are very pathogenic autoimmune antibodies and can on their own bind and then kill neural cells by three mechanisms: excitotoxicity, reactive-oxygen-species (ROS), and complement-fixation. The GluR3**B** peptide antibodies can also induce and/or facilitate brain damage, seizures and various types of behavioral impairments (Levite and Goldberg 2021; Levite 2014; Levite et al. 2020).

**Autoimmune Glutamate receptor antibodies in Nodding syndrome.**We recently published a study on autoimmunity in NS patients that consists of In vitro and In vivo investigations (Levite et al. 2020). NS is a catastrophic type of Epilepsy of unknown etiology, affecting children aged 3–18 years in three sub-Saharan countries including South Sudan, Tanzania and Uganda. The first clinical symptom is often an involuntary nodding of the head in a previously healthy child, potentially triggered by eating. The nodding episodes are thought to be one manifestation of a syndrome that includes various pathological features, among them the following: repetitive head nodding, seizures-usually GCTSs, neurological deterioration and cognitive impairments, stunted growth, weak muscle tone (hypotonia), frequent falling down, breathing problems, wasting and additional problems (Levite et al. 2020; Johnson et al. 2017; Dowell et al. 2013; Colebunders et al. 2023, 2017; Idro et al. 2018; Mwaka et al. 2018; Spencer et al. 2019, 2013, 2022). Eventually, NS leads to death.

In our recent study on 30 South Sudanese epileptic NS patients and healthy subjects (Levite et al. 2020) we found that most NS patients have elevated levels of autoimmune antibodies to three extracellular peptides of Glutamate receptors peptides: AMPA GluR3**B** peptide antibodies (elevated in 86% of patients), NMDA-NR1 peptide antibodies (77%) and NMDA-NR2 peptide antibodies (87%). These Glutamate receptor antibodies found in NS patients (Levite et al. 2020), are the same types of GluR antibodies we found in the serum of IE-3 and IE-15 in the present study.

We further found that the affinity-purified GluR3**B** antibodies of the NS patients by themselves (which we managed to purify from their blood, but not from the Epilepsy patients IE-3 and IE-15 which we investigated in the present study), induced reactive oxygen species (ROS) in human neural cells, and killed these cells in tissue culture (Levite et al. 2020).

Furthermore, video EEG experiments in normal mice, revealed that when the NS patient’s purified IgG antibodies were released continuously (24/7 for 1 week) in naive mouse brain, they induced all the following effects: (1) outburst of seizures, (2) cerebellar purkinje cell loss, (3) degeneration in the hippocampus and cerebral cortex, and (4) elevation of CD3^+^ T cells, and of activated Mac-2^+^ microglia and GFAP^+^ astrocytes in both the gray and white matter of the cerebral cortex, hippocampus, corpus calossum and cerebellum of naïve mice. These effects, induced In vivo by the NS patient’s IgG antibodies in naïve mice (Levite et al. 2020), are in line with the effects induced by the IgG antibodies of the Epilepsy patients IE-3 and IE-15 induced in naïve rats in the present study.

Together, these findings support ‘Autoimmune Epilepsy’ in very different Epilepsy patients.

### Autoimmune GluR3 antibodies can cause profound behavior abnormalities

GluR3**B** antibodies can cause profound brain damage in addition to Epilepsy, including significant changes in recognition memory and impairments in social behavior and in social cognitive functions. This conclusion is based on findings in three different studies.

First, we previously found that GluR3**B** antibodies associate with some cognitive/ psychiatric/behavioral abnormalities in Epilepsy patients (Goldberg-Stern et al. 2014). In that study, we revealed that among the 21 patients with GluR3**B** antibodies, 19 patients (90%) had learning problems, 16 (76%) attention problems, and 15 (71%) psychiatric problems. In contrast, among the 20 patients without GluR3**B**, only 6 (30%) had learning problems (p < 0.0001), 5 (25%) attention problems (p = 0.0017), and 2 (10%) psychiatric problems (p < 0.0001) (Goldberg-Stern et al. 2014).

Second, in another published study we found that GluR3**B**-immunized mice produced high titers of GluR3**B** antibodies, that these mice were significantly more susceptible to seizures, compared to all the groups of control mice, and that the seizure scores associated significantly with the GluR3**B** antibody levels (Ganor et al. 2014), Furthermore, the GluR3**B**-immunized mice were also more anxious in Open-Field test, fell faster in RotaRod test, and fell more in Grip test, compared to all the groups of control mice. Thus, the GluR3**B** antibodies induced abnormal behavior (Ganor et al. 2014).

Third, Scheggia et al. (2021) performed intracerebroventricular infusion of GluR3 antibodies purified from the serum of Frontotemporal Dementia (FTD) patients into mice, and found that the GluR3 antibodies caused a reduction of synaptic levels of GluR3-containing AMPARs in the prefrontal cortex (PFC). In addition, the animals injected with the patient’s GluR3 IgG antibodies showed significant changes in recognition memory and impairments in social behavior and in social cognitive functions. As visualized by confocal imaging, the functional outcomes were paralleled by profound alterations of dendritic spine morphology in the PFC. All the observed behavioral, molecular and morphological alterations were transient and not detected 10–14 days from GluR3 antibodies injection (Scheggia et al. 2021).

### Diagnosis of Glutamate receptor antibodies in Epilepsy patients should be preferably done by testing if patient’s antibodies bind isolated GluR extracellular antigenic peptides

An important diagnosis-related practical fact to note is that in our opinion the most sensitive and reliable In vitro method for detecting autoimmune Glutamate receptor antibodies in blood or CSF of patients seems to be ELISA that tests for binding patient’s antibodies to the GluR3**B** peptide, NR1 peptide and NR2 peptide. It seems that the other methods often fail to detect the autoimmune antibodies to AMPA and NMDA receptors, and the results of the tests are in fact false negative.

### There are many more enigmatic intractable Epilepsy patients who may have pathogenic autoimmune antibodies

Our current study focused only on two intractable and enigmatic epileptic patients IE-3 and IE-15, and revealed a wealth of findings about them, that support the assumption that they suffer from ‘Autoimmune Epilepsy’. As such, the study *cannot* teach us how many more enigmatic Epilepsy patients have similar pathogenic autoimmune antibodies that can on their own induce seizures and neural damage.

However, from the clinical point of view, and as evidenced by Prof. H.G. (2nd author herein) the two Epilepsy patients studied and described in this paper represent a significant proportion (~ 20%) of all the young enigmatic Epilepsy patients treated in the Epilepsy Center, in Schneider Children's Medical Center. In this clinic, ~ 1500 to 2000 Epilepsy patients are diagnosed and treated each year. Therefore, and based on the findings of this paper and other published so far in the field of ‘Autoimmune Epilepsy’, it is important to keep in mind that when an Epilepsy patient has a combination of symptoms including intractable seizures and psychiatric abnormalities, behavioral impairments and some other neurological abnormalities and/or deficits, and a comprehensive work-up (MRI, metabolic) is normal, it is very important to test for autoimmune etiology, by performing all possible In vitro and In vivo tests that can reveal functional active pathological autoimmune antibodies.

The absolute essential tests are the In vitro ones that test the effects of patient’s serum, and preferably purified IgG, on the survival of neural cells.

These functional tests should be done even if the standard passive diagnosis of known autoimmune antibodies yield negative results.

### Appropriate treatments for ‘Autoimmune Epilepsy’ can decrease seizures in intractable patients who have pathological autoimmune antibodies

Patients diagnosed with ‘Autoimmune Epilepsy’ or with encephalitis and seizures, can benefit from various therapeutic strategies, as described and discussed in several original papers and reviews (Dalmau et al. 2019; Britton et al. 2016; Levite and Hart 2002; Quek et al. 2012; Tan et al. 2020; Gao et al. 2016; Dubey et al. 2017, 2018; Bien and Holtkamp 2017; Feyissa et al. 2017, 2018; Cui et al. 2018; Pruss et al. 2012; Greco et al. 2016; Husari and Dubey 2019; Bruijn et al. 2019; Lancaster et al. 2010; Hoftberger et al. 2013; Petit-Pedrol et al. 2014; Carvajal-Gonzalez et al. 2014; Sonderen et al. 2016; Spatola and Dalmau 2017; Finke et al. 2012). Overall, immunotherapy administered early after the first diagnosis of ‘Autoimmune Epilepsy’ seems to be particularly effective, and few optional therapeutic strategies exist, either as a monotherapy or a combinatorial therapy.

These therapeutic options can be classified into three therapeutic groups.

*Group 1: immunosuppressive and anti-inflammatory chemical and biological drugs.*

(A) Intravenous Immunoglobulin (IvIg). IvIg is a ‘cocktail’ product made up of antibodies of thousands of people, to make a super-concentrated and very diverse collection of antibodies against many possible infectious organisms that the body might encounter. IvIg is given intravenously. IvIg can play the role of immunomodulatory and/or even life-saving therapy to modify the course of the underlying disease in patients with autoimmune diseases. (B) Methylprednisolone, (C) Cyclophosphamide, (D) Tacrolimus, (E) Natalizumab—monoclonal antibody (mAb) against the cell adhesion molecules α4-integrins. The α4-integrins are cell surface receptors that mediate cell-extracellular matrix (ECM) and cell–cell adhesions by interacting with fibronectin (FN) and vascular cell adhesion molecule 1 (VCAM-1), respectively. (F) Rituximab—mAb against CD20—a protein expressed primarily on B cells. Anti-CD20 mAbs can act through several mechanisms, including complement-dependent cytotoxicity, antibody-dependent cellular cytotoxicity (ADCC), antibody-dependent cellular phagocytosis, and direct apoptosis induction (for review see Pavlasova and Mraz 2020), (G) Adalimumab—mAb against TNFα. The primary role of TNFα. is in the regulation of immune cells. TNFα induces dozens of effects on target cells that express its receptors.

*Group 2: Immune purging procedures: IgG absorption and plasmapheresis.*

*Group 3: Some AEDs, or a combination of AEDs with immunotherapy.*

Additional therapeutic strategies for ‘Autoimmune Epilepsy’ may be developed in the future, using animal models of this disease, such as the one we developed and used in the present study in rats, or in our previous study in mice (Levite et al. 2020).

### The study limitations

1. This study and its many results are on two Epilepsy patients only. Of course, many more Epilepsy patients need to be investigated in a similar way.
2. Although we found that the IgG antibodies of the two epileptic patients we studied bound to both neural cells of human origin In vitro, in tissue culture (Figs. 5, 6, 7, 8), and to rat neural cells in brains of live rats In vivo (Figs. 11 and 12), these evidences do not prove that the patient’s IgG antibodies bind in the same way to neural cells in their own brains. The direct evidence for that is unattainable, because it is impossible to perform the needed search for such antibodies in the patient’s own brains during their lives.
3. Although we found that IgG antibodies of the two Epilepsy patients we studied, killed both neural cells of human origin In vitro, in tissue culture (Figs. 7 and 8), and rat neural cells in brains of live rats In vivo (Figs. 11 and 12), these evidences do not prove that the patient’s antibodies kill in the same way neural cells in their own brains. The direct evidence for that is also unattainable in the patient’s own brains during their lives.

## Methods

The detailed descriptions of all the methods and specification of the related materials are included in the Supplementary material (due to their very high word counts).

Herein, in the main text of the paper, only four sections are included: (1) study approvals, (2) description of the Epilepsy patients, (3) titles of all the methods, (4) statistical analysis.

### Study approvals

The study on ‘Autoimmune Epilepsy’ in Epilepsy patients has an IRB approval No. 0339-09 in the ethic committee of Rabin Medical Center, which Schneider Medical center is affiliated to. The patients signed informed consent forms. Approval letter of this information, by Prof. Hadassa Goldberg (2nd author), is included in the Supplementary file.

The patients are called throughout the study in coded names: IE-3 and IE-15, and the manuscript does *not* disclose any confidential information about the patients that can identify them.

In addition, the study received approval from the Ethics Committee for Animal Research in the Hebrew University, to conduct the current study in rats (the approval given to Dr. Tawfeeq Shekh-Ahmad, 6th author).

### The intractable Epilepsy patients whose antibodies were investigated in this study

#### Epilepsy patient IE-3

This report on the patient was written by Prof. Hadassa Goldberg, Head of the Epilepsy center at Schneider Children's Medical Center, Israel (second author of this paper). In this clinic, 1500–2000 Epilepsy patients are diagnosed and treated each year.

IE-3 is a 25-year-old girl, born in 1998 after an uneventful pregnancy and normal delivery to healthy parents with no consanguinity. Her motor development was normal. She started to talk only around the age of 3 years and was reported to have some learning difficulties. She attended a normal school and received a lot of special help. There is no family history of seizures or neurological disease**.** Seizures started at the age of 10.5 years, manifested by absences and myoclonic jerks with dropping things from the patient’s hands. The patient had 1–3 GTCGs per month.

Neurological examination at presentation was normal, including normal eye movements, normal fundus and normal cranial nerves. Tone was normal with no weakness. Tendon reflexes were normal. No cerebellar abnormalities were found. Gait was normal.

First electroencephalogram (EEG), shown in Fig. 1, demonstrated mild slowing of the background with generalized discharges of spike wave and polyspikes associated with clinical absences. Brain magnetic resonance imaging (MRI) showed decreased brain parenchyma, with no focal abnormalities. The patient received Valproic acid, an anti-epileptic drug, with partial response. EEG continued to show mild slowing and generalized discharges of spike wave and polyspikes with no photosensitivity.

On examination after 3 months of treatment, there was bilateral dysmetria, action tremor and ataxia of gait. During an attempt to investigate the etiology and characterize the Epilepsy, one of the progressive myoclonic epilepsies was suspected due to a combination of myoclonic Epilepsy with cerebellar signs. Genetic evaluation, including a panel of genes for Epilepsy and exome examination were all negative. In addition, the patient had a detailed metabolic work-up, including blood lactae, pyruvate, amino acids, very long fatty acids, urine for organic acids, lumbar puncture, which were all negative. Muscle biopsy was normal, including staining for mitochondrial disease. Conjunctiva biopsy raised a suspicion of neuronal ceroid liposuscinosis (NCL) deposits, but reexamination of the slides by electron microscopy was negative. A detailed genetic panel for NCL was negative. In 2015, blood and CSF were sent to an external diagnostic clinic for testing if the patient has neuronal autoimmune antibodies directed against: NMDAR, GABARB1, AMPAR1/2 (Glu1/2), CASPR2 and LGI1. None of these autoimmune antibodies were found in the patient’s blood and CSF. The CSF was also negative for anti-neuropil antibodies. In 2019, blood and CSF were sent again to an external diagnostic clinic, for testing the blood neuronal antibodies for NMDR, CASPR2, AMPAR1/2, LGI1, GABAB, Amphiphysin, CV2, PNMA1 (Ma2/Ta), Ri, Yo, Hu, Recoverin, Soxi and Titin. All these were found to be negative again. In the CSF, anti-neuronal antibodies for the same subtypes were also negative. In parallel to the search of autoimmune antibodies in external labs, few serum samples of the patient were sent to the scientific lab of Prof. Mia Levite (last and corresponding author herein) and her research team, at the Faculty of Medicine, The Hebrew University, Jerusalem, Israel, for: 1. Testing whether the patient has specific autoimmune antibodies to three short antigenic peptides of ionotropic glutamate receptors: AMPA-GluR3**B** peptides, NMDA-NR1 and NMDA-NR2, 2. Purifying patient’s IgG from serum, and 3. Testing if the patient’s purified IgG bind and kill human neural cells. The positive results of all these tests are described in this paper. Later, the patient’s purified IgG was studied by Dr. Tawfeeq Shekh-Ahmad and his research team, at The Faculty of Medicine, The Hebrew University, Jerusalem, Israel, for testing In vivo if the patient’s IgG induce seizures and brain damage in naïve rats. The positive results of these In vivo studies are also reported in this paper.

Together, all the findings in this paper indicate that the patient most probably suffers from ‘Autoimmune Epilepsy’, but what caused this disease remains unknown.

Since the patient still had absences and myoclonic jerks and GTCS when treated with Valproic acid, Levetiracetam was added, but led to aggravation of seizures and behavioral problems, and was stopped. Topiramate was tried, but the patient continued to have prolonged absences in clusters. Ethosuximide was added with no relief.

A ketogenic diet was tried, with some improvement. EEG continued to show mild slowing and generalized discharges of spike wave and 3 per second spike wave, some of them associated with clinical manifestation of absence. The patient remained on a ketogenic diet for one year, with combination therapy of Valproic acid, Topiramate and Ethosuximide, with some response but no seizure-freedom. At that point, drop attacks appeared and absences and myoclonic jerks on a daily basis. Levetiracetam was given again.

Vagal nerve stimulator was implanted with no benefit.

The patient cognitive status deteriorated. She stopped attending school and could not walk independently due to severe ataxia and recurrent falls. After the immunological studies raised a suspicion of ‘Autoimmune Epilepsy’, the patient received 3 courses of IVIG (400 mg/kilogram per day for 5 days, once a month) and IV Solumedrol with a low dose of Prednisone. This resulted in some improvement in seizure control, but the general condition deteriorated, and the patient became bedridden with recurrent aspirations.

The family refused a repeat brain MRI because of the need for anesthesia. The family also refused plasmapheresis, for removing circulating autoimmune antibodies. Medical Cannabis (Epidiolex) was tried, to no avail. The patient is currently intubated and bedridden at home.

#### Epilepsy patient IE-15

This report on the patient was written by Prof. Hadassa Goldberg, Head of the Epilepsy center at Schneider Children's Medical Center, Israel (second author of this paper). In this clinic, 1500–2000 Epilepsy patients are diagnosed and treated each year.

IE-15 is a 20-year-old boy, right-handed, with normal perinatal history and no familial history of neurological disorders. No consanguinity. At the age of 11 years, he first presented to the emergency room during an alarming siren in time of war in Israel in 2014, with an event of staring with right-hand movements and right facial twitching, with no loss of sphincter control. The patient was post ictal for about 30 min. In the emergency room, he had two further similar events and was very agitated. The differential diagnosis included an anxiety attack versus seizures. The patient responded to Clonex. His parents described that during the previous year he suffered from anxiety and was in psychological therapy. During hospitalization, the patient had severe behavioral changes with agitation, visual hallucinations, and delirium.

Brain MRI did not show any structural abnormality. CSF examination for signs of inflammation did not find indications for that. PCR for Herpes and other infectious etiologies (including enterovirus, West Nile virus) were all negative. Oligoclonal bands were negative.

Comprehensive diagnostic tests for blood, urine and CSF and genetic analysis are detailed in Table 3. Due to a combination of Epilepsy and psychiatric symptoms, additional tests were taken to rule out Wilson disease, including serum ceruloplasmin, and urine copper, which were normal. No Kayser Fleisher ring on eye examination was documented.

The patient’s serum and CSF were sent to an external diagnostic clinic for testing if the patient has neuronal autoimmune antibodies directed against: NMDAR, AMPA, GABA, CASPR2, LGI1. None of these autoimmune antibodies were found in the patient’s serum and CSF.

In parallel to this search of autoimmune antibodies in external labs, few serum samples of the patient were sent to the scientific lab of Prof. Mia Levite (last and corresponding author herein) and her research team for: 1. Testing whether the patient has specific autoimmune antibodies to 3 antigenic peptides of Glutamate receptors: AMPA-GluR3**B**, NMDA-NR1 and NMDA-NR2, 2. Purifying patient’s IgG from serum, and 3. Testing if the patient’s purified IgG bind and kill human neural cells. The positive results of all these tests are described in this paper. Later, the patient’s purified IgG was studied by Dr. Tawfeeq Shekh-Ahmad and his research team for testing In vivo if the patient’s purified IgG induce seizures and brain damage in naïve rats. The positive results of these in vivo studies are also reported in this paper.

Together, all the findings in this paper indicate that the patient most probably suffers from ‘Autoimmune Epilepsy’, but what caused this disease remains unknown.

The seizures were controlled with Carbamazepine.

After the diagnosis of ‘Autoimmune Epilepsy’ was made, the patient received IV Solumedrol 30 mg per kilogram for 5 days, with behavioral improvement. IVIG did not improve the patient’s condition. The patient was discharged on a combination therapy of Carbamazepine and Resperidal and returned to a regular school with follow-up appointments at the Epilepsy and psychiatry clinics. EEG a month later still showed some slowing with no epileptiform activity. Brain MRI demonstrated mild brain atrophy, which was attributed to the steroid treatment.

In 2016, then aged 13 years-old, the patient was readmitted following a GTCS during a febrile illness after seizure freedom for more than 2 years. EEG showed mild slowing with no epileptiform activity. Lacosamide was added to his antiepileptic therapy. Repeated lumbar puncture and subsequent tests in external clinic for the presence of autoimmune and paraneoplastic antibodies did not reveal evidence for the presence of these antibodies, or for an infectious cause. Video EEG in 2022, whilst tapering Carbamazepine, documented one focal seizure with right posterior temporal onset. The patient’s EEG, shown in Fig. 1, and the repeated high resolution MRI did not demonstrate any structural abnormality.

In summary, this patient suffers from intractable Epilepsy with psychiatric symptoms including visual hallucinations with wax and wane course since age 11 years. His current medication consists of a combination of Carbamazepine. Lamotrigine and Lacosamide with Lustral and Arply. He still has brief focal seizures (3–4 per month) with no hallucinations or delusions, and he is able to attend his studies in School. In addition, low cerebral folate was noted in the CSF (which may be secondary to Carbamazepine therapy) and the patient received daily Leucoverin. However, each attempt to taper down the Carbamazepine has led to seizure relapse.

### Purification of IgG from serum of Epilepsy patients and healthy subjects

The detailed description of this method is written in the ‘Supplementary material’.

### Detection by ELISA of autoimmune antibodies directed against extracellular antigenic of ionotropic glutamate receptor peptides: AMPA GluR3B peptide, NMDA-NR1 peptide and NMDA-NR2 peptides

The detailed description of this method is written in the ‘Supplementary material’.

### The human neural cells used in the study

The detailed description of this method is written in the ‘Supplementary material’.

### Immunofluorescence staining and confocal microscopy for detecting the binding of IgG antibodies of Epilepsy patients to human neural cells

The detailed description of this method is written in the ‘Supplementary material’ in vitro.

### Immunofluorescence staining and confocal microscopy for detecting the In vitro killing of live human neural cells by IgG antibodies of Epilepsy patients

The detailed description of this method is written in the ‘Supplementary material’.

### The antibodies used for immunofluorescence staining and microscopy experiments

The detailed description of this method is written in the ‘Supplementary material’.

### Detection of seizures induced In vivo in normal rats by Epilepsy patient's IgG antibodies released contentiously in the brain, using continuous EEG recording electrodes and video photography

The detailed description of this method is written in the ‘Supplementary material’.

### Immunohistochemistry and microscopy of rat brain sections

The detailed description of this method is written in the ‘Supplementary material’.

### Statistical analysis

1. Statistical analysis of the findings related to the levels of antibodies in the serum of the Epilepsy patients antibody and their In vitro effects on human neural cells.

The statistical analysis of all the In vitro experimental data allowing statistical analysis that included: determination of the levels of IgG antibodies and Glutamate receptor antibodies, and the extent of binding and killing of human neural cells by Epilepsy patient’s IgG antibodies was performed by T-test.

2. Statistical analysis related to analysis of the seizure induced by Epilepsy patient’s IgG antibodies in rat brain In vivo. The statistical analysis employed in the experimental data for animals with or without seizures (Fig. 9G), cumulative no of seizures (Fig. 9H), and seizure duration (Fig. 10F) includes, a Chi-square test, One-way Anova followed by Dunnett's multiple comparison and T-test respectively and where applicable. Differences with *p < 0.05, **p < 0.01, ***p < 0.001 were considered statistically significant.

3. Statistical analysis related to analysis of the binding and killing in vivo of Epilepsy patient’s IgG antibodies to neurons and astrocytes in rat brain.

All the data concerning the analysis of the binding and killing of neurons and astrocytes by patient's IgG antibodies was subjected to one-way ANOVA followed by Dunnett's multiple comparison test. Data are displayed as the mean ± SEM, differences with *p < 0.05, **p < 0.01, ***p < 0.001 were considered statistically significant.

## Supplementary Information

Below is the link to the electronic supplementary material.

Supplementary file1 (DOCX 113 KB)

## Data Availability

The manuscript has no associated data.
