# Supplementary material for: Enigmatic intractable Epilepsy patients have antibodies that bind glutamate receptor peptides, kill neurons, damage the brain, and cause Generalized Tonic Clonic Seizures
Source: J Neural Transm (Vienna). 2025 Feb 11;132(5):663–88. doi: 10.1007/s00702-024-02855-2 (PMC12043744; doi:10.1007/s00702-024-02855-2)

SUPPLEMENTARY MATERIAL

**Materials and Methods (Part 4 of the manuscript)**

**4.1 Study Approvals**

The study on Autoimmune Epilepsy in epilepsy patients has an IRB approval No. 0339-09 in the ethic committee of Rabin Medical Center, which Schneider Medical center is affiliated to. The patients signed informed consent forms. Approval letter of this information, by Prof. Hadassa Goldberg (3^rd^ author), is included in the Supplementary file.
The patients are called throughout the study in coded names: IE-3 and IE-15, and the manuscript does **not** disclose any confidential information about the patients that can identify them.

In addition, the study received approval from the Ethics Committee for Animal Research in the Hebrew University, to conduct the current study in rats (the approval given to
Dr. Tawfeeq Shekh-Ahmad (6^th^ author).

**4.2 The Intractable Epilepsy patients whose antibodies were investigated in this study**

**4.2.1 Epilepsy patient IE-3**

This report on the patient was written by Prof. Hadassa Goldberg, Head of the epilepsy center at Schneider Children's Medical Center, Israel (and third author of this paper). In this clinic, 1500-2000 epilepsy patients are diagnosed and treated each year.

IE-3 is a 25-year-old girl, born in 1998 after an uneventful pregnancy and normal delivery to healthy parents with no consanguinity. Her motor development was normal. She started to talk only around the age of 3 years and was reported to have some learning difficulties. She attended a normal school received a lot of special help. There is no family history of seizures or neurological disease**.** Seizures started at the age of 10.5years, manifested by absences and myoclonic jerks with dropping things from the patient’s hands.
The patient had 1-3 GTCGs per month.
Neurological examination at presentation was normal, including normal eye movements, normal fundus and normal cranial nerves. Tone was normal with no weakness. Tendon reflexes were normal. No cerebellar abnormalities were found. Gait was normal.

First electroencephalogram (EEG), shown in Suppl. Fig. 1), demonstrated mild slowing of the background with generalized discharges of spike wave and polyspikes associated with clinical absences. Brain magnetic resonance imaging (MRI) showed decreased brain parenchyma, with no focal abnormalities. The patient received Valproic acid, an anti-epileptic drug, with partial response. EEG continued to show mild slowing and generalized discharges of spike wave and polyspikes with no photosensitivity.
On examination after 3 months of treatment, there was bilateral dysmetria, action tremor and ataxia of gait. During an attempt to investigate the etiology and characterize the epilepsy one of the progressive myoclonic epilepsies was suspected due to a combination of myoclonic epilepsy with cerebellar signs. Genetic evaluation, including a panel of genes for epilepsy and exome examination were all negative. In addition, the patient had a detailed metabolic work-up, including blood lactae, pyruvate, amino acids, very long fatty acids, urine for organic acids, lumbar puncture, which were all negative. Muscle biopsy was normal, including staining for mitochondrial disease. Conjunctiva biopsy raised a suspicion of neuronal ceroid liposuscinosis (NCL) deposits, but reexamination of the slides by electron microscopy was negative. A detailed genetic panel for NCL was negative. In 2015, blood and cerebrospinal fluid (CSF) was sent to an external diagnostic lab in another hospital for testing if the patient has neuronal autoimmune antibodies directed against: NMDAR, GABARB1, AMPAR1/2 (Glu1/2), CASPR2 and LGI1. None of these autoimmune antibodies were found in the patient’s blood and CSF. In the CSF, The CSF was also negative for anti-neuropil antibodies. In 2019, blood and CSF were sent again to an external diagnostic lab in another hospital, for testing the blood neuronal antibodies for NMDR, CASPR2, AMPAR1/2, LGI1, GABAB, Amphiphysin, CV2, PNMA1 (Ma2/Ta), Ri, Yo, Hu, recoverin, soxi and titin. All these were found to be negative again. In the CSF, anti-neuronal antibodies for the same subtypes were also negative. In parallel to the search of autoimmune antibodies in external labs, few serum samples of the patient were sent to the scientific lab of Prof. Mia Levite (second author herein) and her research team, at the Faculty of Medicine, The Hebrew University, Jerusalem, Israel, for: 1. Testing whether the patient has specific autoimmune antibodies to three short antigenic peptides of ionotropic glutamate receptor antibodies: AMPA-GluR3B peptides, NMDA-NR1 and NMDA-NR2, 2. Purifying patient’s IgG from serum, and 3. Testing if the patient’s purified IgG bind and kill human neural cells. The positive results of all these tests are described in this paper. Later, the patient’s purified IgG was studied by Dr. Tawfeeq Shekh-Ahmad and his research team, at The Faculty of Medicine, The Hebrew University, Jerusalem, Israel, for testing in vivo ability if the patient’s IgG induce seizures and brain damage in naïve rats. The positive results of these in vivo studies are also reported in this paper.

Together, all the findings in this paper indicate that the patient most probably suffers from Autoimmune Epilepsy, but what caused this disease remains unknown.

Since the patient still had absences and myoclonic jerks and generalized tonic clonic seizures when treated with Valproic acid, Levetiracetam was added, but led to aggravation of seizures and behavioral problems, and was stopped. Topiramate was tried, but the patient continued to have prolonged absences in clusters. Ethosuximide was added with no relief.

A ketogenic diet was tried, with some improvement. EEG continued to show mild slowing and generalized discharges of spike wave and 3 per second spike wave, some of them associated with clinical manifestation of absence. The patient remained on a ketogenic diet for one year, with combination therapy of Valproic acid, Topiramate and Ethosuximide, with some response but no seizure-freedom. At that point, drop attacks appeared and absences and myoclonic jerks on a daily basis. Levetiracetam was given again.

Vagal nerve stimulator was implanted with no benefit.

The patient cognitive status deteriorated. She stopped attending school and could not walk independently due to severe ataxia and recurrent falls. After the immunological studies raised a suspicion of Autoimmune Epilepsy, the patient received 3 courses of IvIg (400 mg/kilogram per day for 5 days, once a month) and IV Solumedrol with a low dose of prednisone. This resulted in some improvement in seizure control, but the general condition deteriorated, and the patient became bedridden with recurrent aspirations.

The family refused a repeat brain MRI because of the need for anesthesia. The family also refused plasmapheresis, for removing circulating autoimmune antibodies. Medical Cannabis (Epidiolex) was tried, to no avail. The patient is currently intubated and bedridden at home.

**4.2.2 Epilepsy patient IE-15**

This report on the patient was written by Prof. Hadassa Goldberg, Head of the epilepsy center at Schneider Children's Medical Center, Israel (and third author of this paper). In this clinic, 1500-2000 epilepsy patients are diagnosed and treated each year.

IE-15 is a 20-year-old boy, right-handed, with normal perinatal history and no familial history of neurological disorders. No consanguinity. At the age of 11 years, he first presented to the emergency room during an alarming siren in time of war in Israel in 2014, with an event of staring with right-hand movements and right facial twitching, with no loss of sphincter control. The patient was post ictal for about 30 minutes. In the emergency room, he had two further similar events and was very agitated. The differential diagnosis included an anxiety attack versus seizures. The patient responded to Clonex. His parents described that during the previous year he suffered from anxiety and was in psychological therapy. During hospitalization, the patient had severe behavioral changes with agitation, visual hallucinations, and delirium.

Brain MRI did not show any structural abnormality. CSF examination for signs of inflammation did not find indications for that. PCR for Herpes and other infectious etiologies (including enterovirus, West Nile virus) were all negative. Oligoclonal bands were negative.

Comprehensive diagnostic tests for blood, urine and CSF and genetic analysis are detailed in Table 3. Due to a combination of epilepsy and psychiatric symptoms, additional tests were taken to rule out Wilson disease, including serum ceruloplasmin, and urine copper, which were normal. No Kayser Fleisher ring on eye examination was documented.

The patient’s serum and CSF were sent to an external diagnostic lab in another hospital for testing if the patient has neuronal autoimmune antibodies directed against:

NMDAR, AMPA, GABA, CASPR2, LGI1. None of these autoimmune antibodies were found in the patient’s serum and CSF.

In parallel to this search of autoimmune antibodies in external labs, few serum samples of the patient were sent to the scientific lab of Prof. Mia Levite (second author herein) and her research team for: 1. Testing whether the patient has specific autoimmune antibodies to 3 antigenic peptides of glutamate receptor antibodies: AMPA-GluR3B peptides, NMDA-NR1 and NMDA-NR2, 2. Purifying patient’s IgG from serum, and 3. Testing if the patient’s purified IgG bind and kill human neural cells. The positive results of all these tests are described in this paper. Later, the patient’s purified IgG was studied by Dr. Tawfeeq Shekh-Ahmad and his research team for testing in vivo if the patient’s purified IgG induce seizures and brain damage in naïve rats. The positive results of these in vivo studies are also reported in this paper.

Together, all the findings in this paper indicate that the patient most probably suffers from Autoimmune Epilepsy, but what caused this disease remains unknown.

The seizures were controlled with Carbamazepine.

After the diagnosis of autoimmune epilepsy was made, the patient received IV solumedrol 30 milligram per kilogram for 5 days, with behavioral improvement. IVIG did not improve the patient’s condition. The patient was discharged on a combination therapy of Carbamazepine and Resperidal and returned to a regular school with follow-up appointments at the epilepsy and psychiatry clinics. EEG a month later still showed some slowing with no epileptiform activity. Brain MRI demonstrated mild brain atrophy, which was attributed to the steroid treatment.

In 2016, then aged 13 years-old, the patient was readmitted following a generalized tonic clonic seizure during a febrile illness after seizure freedom for more than 2 years. EEG showed mild slowing with no epileptiform activity. Lacosamide was added to his antiepileptic therapy. Repeated lumbar puncture and subsequent tests in external lab in another hospital for the presence of autoimmune and paraneoplastic antibodies did not reveal evidence for the presence of these antibodies, or for an infectious cause. Video EEG in 2022, whilst tapering Carbamazepine, documented one focal seizure with right posterior temporal onset. The patient’s EEG, shown in Fig. 2, and the repeated high resolution MRI did not demonstrate any structural abnormality.
In summary, this patient suffers from intractable epilepsy with psychiatric symptoms including visual hallucinations with wax and wane course since age 11 years. His current medication consists of a combination of Carbamazepine. Lamotrigine and Lacosamide with Lustral and Arply. He still has brief focal seizures (3-4 per month) with no hallucinations or delusions, and he is able to attend his studies in School. In addition, low cerebral folate was noted in the CSF (which may be secondary to Carbamazepine therapy) and the patient received daily Leucoverin. However, each attempt to taper down the Carbamazepine has led to seizure relapse.

**4.3 Purification of IgG from serum of epilepsy patients and healthy subjects**

Sera of 15 intractable epilepsy patients: IE-3 and IE-15 and 13 others, all treated by Prof. Hadassah Goldberg at the Schneider Children's Medical Center, Israel, and of 9 healthy subjects was sent for IgG purification at a professional protein lab, at the Wolfson center of Applied Structural Biology, in the Hebrew University, Jerusalem, Israel. There, the sera of all the epilepsy patients and healthy subjects were centrifuged at 1600 RPM for 10 min 4^0^C. Clean supernatants were diluted to final 4ml with PBS (10mM NaPO_4_, 150mMNaCl pH7.4). Sample was loaded onto a PBS equilibrated 1ml Protein G Sepharose FF (GE Healthcare) affinity chromatography column using AKTA AVANT (GE Healthcare). Then the column was washed with PBS until reaching low OD280nm; IgG was eluted with 0.1M Glycine pH3.0 buffer and 1ml eluted fractions were collected in tubes containing 0.08ml 1.5M Tri-HCl pH 8.5 to get immediate pH neutralization. IgG fractions were pool according to OD280nm profile and dialyzed 3hrs vs 100ml PBS and ON 4°C using fresh PBS (using 13kDa cutoff dialyze tubes. Finally, the sera was concentrated by ultrafiltration devices (cut-off 30kDa of Amicon), and the purified IgG samples were analyzed for contents by A280 (E.C 1.35) and by SDS-PAGE (Novex 4-12%, Thermo), using reduced and non-reduced sample buffer. PageRuler (Thermo) was used as a Molecular Weight (MW) marker. The purified IgG samples were aliquoted and frozen at -80C Later, frozen IgG samples were thawed and used for all the functional studies reported in this manuscript.

**4.4 Detection by ELISA of autoimmune antibodies directed against extracellular antigenic of ionotropic glutamate receptor peptides: AMPA GluR3B peptide, NMDA-NR1 peptide and NMDA-NR2 peptides**

The Glutamate receptor peptides used in the ELISA for detection of the respective autoimmune antibodies
Three **extracellular antigenic peptides of ionotropic glutamate receptor subunits** were synthesized in an external professional lab which specializes in synthesis and purification of peptides (Thermo Fisher Scientific, USA). These peptidesIncluded: [1] AMPA-GluR3B peptide - a 24 amino acid peptide, whose amino acid (aa) sequence is NEYERFVPFSDQQISNDSSSSENR[), corresponding to aa372](file:///C:\Users\owner\Desktop\AUTOIMMUNE%20EPILEPSY%20-%20talks,%20papers,exp\??.htm)-[395 of the glutamate/AMPA receptor GluR3](file:///C:\Users\owner\Desktop\AUTOIMMUNE%20EPILEPSY%20-%20talks,%20papers,exp\??.htm); [2] [NMDA-NR1](file:///C:\Users\owner\Desktop\AUTOIMMUNE%20EPILEPSY%20-%20talks,%20papers,exp\??.htm) peptide - a 15aa peptide, whose aa sequence is TEKPRGYQMSTRLK, [corresponding to extracellular N-terminus aa residues 385-399 of NMDA](file:///C:\Users\owner\Desktop\AUTOIMMUNE%20EPILEPSY%20-%20talks,%20papers,exp\??.htm) Receptor 1; and [3] [NMDA-NR2](file:///C:\Users\owner\Desktop\AUTOIMMUNE%20EPILEPSY%20-%20talks,%20papers,exp\??.htm)A peptide - a 15aa peptide, whose aa sequence is SVSYDDWDYSLEARV, corresponding to extracellular N-terminus aa residues aa 278292 [of the NMDA-R2A subunit of NMDA](file:///C:\Users\owner\Desktop\AUTOIMMUNE%20EPILEPSY%20-%20talks,%20papers,exp\??.htm) receptor.

ELISA for detection of specific antibodies directed against the AMPA-GluR3B peptide, NMDA-NR1 peptide, and NMDA-NR2 peptide

Sera of the epilepsy patients and healthy individuals were tested simultaneously and in a blinded fashion for GluR3B, NR1 and NR2 antibodies by ELISA, as done previously in several of our studies (see for example ([1](#_ENREF_1))). In the first (coating) step, microtiter wells of three Maxisorp microtiter immunoplates (Nunc, Roskilde, Denmark) were covered with 50 µL per well of 10^_7^ M of either GluR3**B** peptide (1^st^ plate), NR1 peptide (2^nd^ plate), or NR2 peptide (3^rd^ plate), suspended in a coating buffer of 0.1 M NaHCO3 pH 8.2 for detecting the respective antibodies. In parallel, the microtiter wells of a 4^th^ control microtiter plate were covered with 50 μl per well of phosphate buffered saline (PBS) with 1% bovine serum albumin (BSA) only (i.e. without any antigenic peptide), to detect nonspecific control binding to BSA.

The four microtiter plates were incubated overnight at 4^o^C refrigerator.
The following day, the microtiter wells of each of the four plates were washed twice with 100 ml of PBS with 1% BSA per well, and then once with 100 μl per well of double distilled water (DDW). The next step was the blocking of the non-specific binding, performed by adding 50 μl per well of PBS with 1% BSA to all the microtiter wells, and subsequent 2 h incubation at room temperature. At the end of the blocking stage, the microtiter wells were washed, first with PBS with 1% BSA and then with DDW, as in the previous washing step.. Thereafter, the serum of each patient or control individual underwent serial dilutions in PBS with 1% BSA, to final dilutions of 1:10, 1:100, and 1:1000. Then, 100 μof each serum at each of the 3 dilutions was added to two adjacent duplicate microtiter wells, in each of the four plates: the GluR3B-, NR1-, NR2-, PBS+BSA-coated, plates. All the microtiter plates were then incubated overnight at 4^o^C refrigerator. The next day, the wells were washed once more, first with PBS with 1% BSA and then with DDW, as in the previous washing steps. Thereafter, Horseradish peroxidase (HRP)-conjugated goat anti-human IgG (Jackson Immunoresearch, West Grove, PA) was diluted in PBS with 1% BSA to a final dilution of 1:1000, and 50 μl of this dilution was added to each well in the four microtiter plates. Then, the plates were incubated for 2 h at room temperature. Following additional washing step, as described above, ABTS peoxidase substrate (Kirkegaard & Perry Laboratories, Gaithersburg, MD) was added to each well, and the optical density (OD) at 405 nm was measured with an ELISA reader. Repeated OD measurements of the plates were performed 3-4 times, at approximately 20 min intervals, from the time the substrate was added. The first OD reading was done when the green color in some wells became clear.

To obtain the level of the specific GluR3B peptide antibodies, NR1 peptide antibodies, and NR2A peptide antibodies, in each serum, a two-step calculation was performed for each serum dilution, of each epilepsy patient or healthy individual, for each immunoplate, and for each OD reading (1st, 2nd, 3rd or 4th), as follows:

The two-step calculation included: (1) calculation of the average value of every set of adjacent duplicate wells; (2) subtraction of the average OD of the nonspecific binding to PBS/BSA of each serum sample, in each dilution and each OD reading, from the average OD of the specific binding of that specific serum to either GluR3B peptide, or NR1 peptide, or NR2 peptide, of the very same serum sample, at the same dilution, and the same OD reading.

Thus, the final values of specific GluR3B peptide antibodies, NR1 peptide antibodies, and NR2A antibodies were determined for each serum dilution using the equation: [average OD of duplicate wells in the GluR3B-coated or NR1-cotaed or NR2-coated plate] - [average OD of duplicate wells in the PBS/BSA-coated plate]. In most cases, the results of the second OD reading (~30 min after adding the substrate) were the clearest.

Findings were considered positive if the final value of specific GluR3B peptide antibodies, NR1 peptide antibodies, and NR2A antibodies was equal or higher than the average OD + 2 standard deviation (SD) of the average of all the healthy individuals, tested in the same assay (used as cutoff), in the same microtiter plates. In most experiments, the calculated cutoff was approximately 0.25—0.4 OD.

**4.5 The human neural cells used in the study**

The human neural cells used in this study in several experiments, to test the binding and killing of the cells by the IgG of the epilepsy patients, were grown and differentiated from human human embryonic stem cells (hESCs), as previously described ([2](#_ENREF_2)), ([3](#_ENREF_3), [4](#_ENREF_4)), and used successfully in various previous studies. In brief, the neural differentiation

was performed based on the published protocols, with slight modifications: The hESCs were grown on mitomycin-c treated Foreskin cells and were detached after 1 week with Collagenase IV (Worthington #LS004152). The floating clusters were grown for 3-4 weeks with bFGF 20 ng/ml (Peprotech) and LDN193189 100nM (Stemgent). SB431542F 5uM (Cayman Chemical), was added to the medium in the first 3 days. For further differentiation and maturation, the neural spheres were dissociated to single cells/small clumps using TrypLe Select (Gibco) and were seeded on glass coverslips, pretreated with Poly-d-lysine, Laminin and Fibronectin (all from Sigma). The human cells were then incubated with the same medium sup with: BDNF (10ng/ml), GDNF (10ng/ml), dbcAMP (1uM),AA (10ng/ml) with addition of ROCK inhibitor (10uM) only for overnight. The human cells were then incubated for 6 more days, then were split using Accutaze, and finally seeded on new cover slips for 4-6 more days. The slides containing the human neural cells were then used for the various experiments described in this paper.

**4.6 Immunofluorescence staining and confocal microscopy for detecting the In vitro binding of IgG antibodies of epilepsy patients to human neural cells**

Human neural cells were treated along the following steps (and 3 washes were performed in between key steps): **1.** Fixation with 4% paraformaldehyde; **2**. Incubation with either the serum (1:10 or 1:30), or total IgG (~0.2 mg/ml= dilution of 1:10 of the original IgG yield after IgG purification, which was ~2mg/ml), or affinity-purified antibodies of NS patients or healthy subjects (either 1hr in RT, or ON in 4C); **3**.Blocking with PBS 2% BSA (30 min RT);
**4**. Incubation with a secondary Cy3- Goat and human F(ab)_2_ fragment (30min RT); **5**. Permeabilization and further blocking (0.2% Triton, 2% BSA in PBS, 30 min, RT); **6**. For staining neurons, the cells were with mouse anti-human β3 antibody. **7**. Incubation with a secondary antibody: either goat anti-mouse Alexa 647 antibody (1:100, 30 min, RT), or anti-mouse-FITC antibody (1:100, 30 min, RT). DAPI (1:1000; DAPI, dilactate, Invitrogen), for staining the cell's nuclei, was added in this step too.

In all binding experiments, the human neural cells were fixed before addition of the human IgG, for preserving the intact ultrastructure of the neural cells, and optimal binding of the IgG to the cellular components.

**4.7 Immunofluorescence staining and confocal microscopy for detecting the In vitro killing of live human neural cells by IgG antibodies of epilepsy patients**

Human neural cells (grown as described above) were treated along the following steps (and 3 washes were performed in between key steps): **1**. Incubation with either the serum (1:10), or total IgG (~0.2 mg/ml=, or affinity-purified antibodies of NS patients or healthy subjects (either 1hr in RT, or ON in 4C); **2.** Blocking with PBS 2% BSA (30 min RT); **3.** For staining dead cells: Incubation with Sytox green (ThremoFisher Scientific' 1micromolar, 30 min, RT); **4.** Fixation with 4% paraformaldehyde; **5**. Blocking with PBS 2% BSA (30 min RT); **6.** Incubation with a secondary Cy3- Goat and human F(ab)2 fragment (Jackson ImmunoResearch code: 109-166-088) (30min RT):**7**. Permeabilization and further blocking (0.2% Triton, 2% BSA in PBS, 30 min, RT); **8**. For staining neurons: Incubation with mouse anti-human β For staining neurons: Incuba **9**. Incubation with a secondary antibody: either goat anti-mouse Alexa 647 antibody (1:100, 30 min, RT), or anti-mouse-FITC antibody (1:100, 30 min, RT). DAPI 1:1000), for staining the cell's nuclei, was added in this step too.

**4.8 The antibodies used for immunofluorescence staining and microscopy experiments**

Cy3- Goat and human F(ab)2 fragment - Cat no: 109-166-088; Cy5- Donkey anti Rabbit IgG (H+L) (Jackson Cat.# 711-175-152); Cy5- Donkey Anti-Mouse IgG (H+L) Jackson Cat.# 715-175-151; Alexa Fluor 488 Donkey Anti Rabbit  F(ab') Fragment IgG (H+L) - Jackson Cat.# 711-546-152; Alexa Fluor 647 Goat Anti Mouse  IgG (H+L) - Jackson Cat.# 115-605-003; FITC Goat Anti Mouse IgG - DAKO Cat.#F0479; Monoclonal Mouse Anti Human-β-Tubulin Isotype III IgG2b - Sigma T5076; Rabbit Polyclonal Anti Human - GFAP STEM CELL TECHNOLOGIES Cat.#1439; Mouse anti-GluR3**B** monoclonal antibody - Medimab Cat.# GLU149.29.61.

**4.9 Detection of seizures induced In vivo in normal rats by epilepsy patient's IgG antibodies released contentiously in the brain, using continuous EEG recording electrodes and video photography**

Animals and surgery. The animal experiments followed the guidelines set by the Association for Assessment and Accreditation of Laboratory Animal Care International and received approval from the Institutional Animal Care and Use Committee of the Hebrew University of Jerusalem (Approval number: MD-20-16254-5). In this study naive male and female Sprague-Dawley (SD) rats (180-220 g), which were a strain of the Hebrew University, were obtained from Harlan in Jerusalem, Israel. The rats were kept under controlled environmental conditions with a temperature of 23C, 50-60% humidity, and a 12-hour light/dark cycle. They had access to food and water without any restrictions. The rats were allowed to acclimatize for 7 days before the beginning of the experiment.

SD rats (200-250 g) were anesthetized using isoflurane (3% induction, 1.5–2% for surgery) and fixed to the stereotaxic frame (Kopf, CA, USA). Prior to initiating surgery, rats were injected with buprenorphine (0.2 mg/kg; SC) and Metacam (1 mg/kg; SC) for pain relief. A subcutaneous ECoG transmitter (A3028E, Open-Source Instruments) with two subdural intracranial electrodes was implanted (Staba et al., 2017). The recording electrode was placed above the right hippocampus at 2.5 mm lateral and 4 mm posterior of bregma ([5) (Paxinos, 2014 #1628](#_ENREF_5)). The reference electrode was implanted on the contralateral hemisphere at 2.5 mm lateral and 6 mm posterior of bregma ([5) (Paxinos, 2014 #1628](#_ENREF_5)). The electrodes were affixed to the skull using three skull screws and tissue glue. In addition, a brain infusion cannula (Brain Infusion Kit 2, Alzet) was implanted into the right lateral ventricle of the brain at 0.7 mm posterior, 1.8 mm lateral, 4.5 mm ventral from the bregma (Paxinos and Watson, 2014). The catheter tube was plugged for later connection to a mini osmotic pump, and a tunnel was created to position the tube into a subcutaneous cavity. After surgery, the brain incision was sealed with dental cement, and the rats were injected with 5ml glucose and 3-5 mL and amoxicillin (Betamox LA, 100 mg/kg).

Subcutaneously implanted osmotic minipump pre-loaded with 200µl serum purified IgG (1mg/ml) derived from epilepsy patients and their control counterparts was continuously infused into the brain at a release rate of 1µl per hour. These rats were monitored for seizure activity, using wireless ECoG and video recording connected to telemetry setup integrated with CCTV, for 4 weeks. EEG recorded data were analyzed for seizure activity, and other EEG impairments. At the end of experiment, rats were sacrificed, perfused, and brain tissue samples were collected.

Collected brain samples were subjected to further immunohistochemical analysis including binding of patient derived antibodies to the various brain regions and examine brain damages induced by patient-derived IgG(s).

**4.10 Immunohistochemistry and microscopy of rat brain sections**Animals were perfused transcardially with cold heparin+ PBS (to get rid of blood in the brain) then they were perfused with 4% PFA prior to removal of the whole brain. Brain Tissues were then fixed in fresh 4% paraformaldehyde for 24hrs post perfusion. Brain tissues were cryoprotected by placement in 15% sucrose in PBS until tissue sinks (6-12hrs) and then 30% sucrose in PBS for overnight or until tissue sinks. After cryoprotection, brains were embedded in O.C.T. Compound (Scigen) and stored at -80^0^C. For each animal, coronal sections (20µm) selected from the hippocampus were cut in a cryostat (Leica CM1950) at -20^0^C and fixed on poly-L-lysine coated slides (Thermo Fisher Scientific), then left to air dry at room temperature for 2 h. Brain sections were circled with a water repellent pen (Dako pen; Agilent), permeabilized with PBS, 0.2% Triton X-100 (Sigma) for 30 min, blocked with 4% goat and donkey serum (Sigma) for 2–3 h then washed three times for 10 min each with PBS. Sections were incubated overnight at 4^0^C with a rabbit primary antibody against NeuN (1:500, ab177487), GFAP (1:500, ab4674) (Abcam, Cambridge, UK) in a solution of PBS, 0.1% triton X-100 and BSA 1%. Following three washes with PBS (10 min each); the sections were incubated with Alexa Fluor® 488 goat anti-rabbit secondary antibody (1:500; ab150081), Alexa Fluor® 647 goat anti-chicken secondary antibody (1:500; ab150081, Abcam, Cambridge, UK), anti-human IgG Cy3-conjugated Streptavidin (1:200, Jackson) for 2 h at room temperature and kept in dark. The sections were washed three times with PBS (10 min each) and mounted with Vectashield and 40,6-diamidino-2-phenylindole (DAPI) mounting medium (Vector Labs, Burlingame, CA, USA). Images were obtained at a resolution of 1024x1024 on Nikon confocal A1R microscope using a 20X objective. Images were acquired at 405 nm excitation wavelength and 455 nm emission wavelength for DAPI, excitation of 488 nm and emission of 520 nm for NeuN, excitation of 647 nm and emission of 670 nm for GFAP and excitation of 520 nm and emission of 570 nm for CY3. Image analysis was performed using ImageJ software.

**4.11 Statistical analysis
1. Statistical analysis of the findings related to the levels of antibodies in the serum of the epilepsy patients antibody and their In vitro effects on human neural cells**.
The statistical analysis of all the In vitro experimental data allowing statistical analysis that included: determination of the levels of IgG antibodies and glutamate receptor antibodies, and the extent of binding and killing of human neural cells by epilepsy patient’s IgG antibodies was performed by T-test.

**2. Statistical analysis related to analysis of the seizure induced by epilepsy patient’s IgG antibodies in rat brain in vivo.** The statistical analysis employed in the experimental data for Animals with or without seizure (Fig. 9G), Cumulative no of seizures (Fig. 9H), and seizure duration (Fig10 F) includes, a Chi-square test, One-way Anova followed by Dunnett's multiple comparison and T-test respectively and where applicable. Differences with * p < 0.05, ** p < 0.01, *** p < 0.001 were considered statistically significant.

**3. Statistical analysis related to analysis of the binding and killing in vivo of epilepsy patient’s IgG antibodies to neurons and astrocytes in rat brain.**

All the data concerning the analysis of the binding and killing of neurons and Astrocytes by patient's IgG antibodies was subjected to one-way ANOVA followed by Dunnett's multiple comparison test. Data are displayed as the mean ±SEM, differences with * p < 0.05, ** p < 0.01, *** p < 0.001 were considered statistically significant.

**Study approval letter**


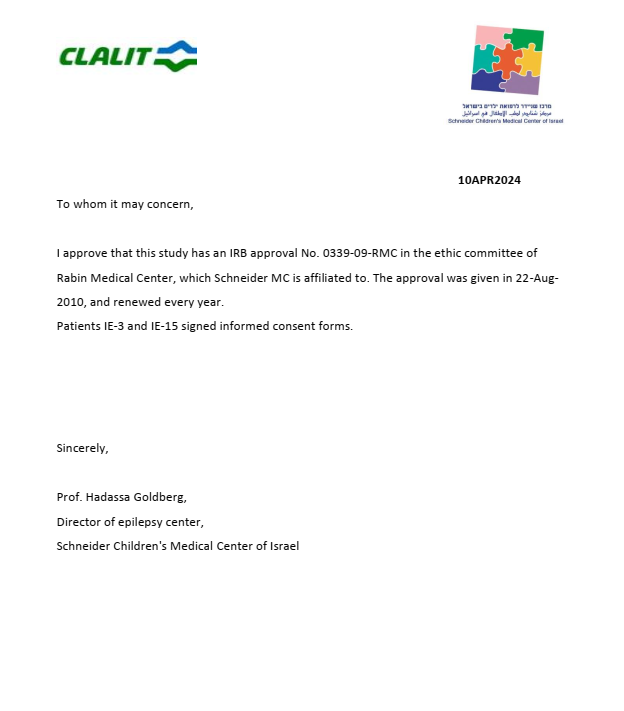

Supplement: Supplementary file 1 — Supplementary file1 (DOCX 113 KB) [file 702_2024_2855_MOESM1_ESM.docx]
